# Essentials in saline pharmacology for nasal or respiratory hygiene in times of COVID-19

**DOI:** 10.1007/s00228-021-03102-3

**Published:** 2021-03-27

**Authors:** Suzy Huijghebaert, Levi Hoste, Guido Vanham

**Affiliations:** 1 Independent Research Support, La Hulpe, Belgium; 2 Pediatric Pulmonology, Infectious Diseases and Immunology, Ghent University Hospital, Ghent, Belgium; 3 Primary Immunodeficiency Research Lab, Center for Primary Immunodeficiency Ghent, Jeffrey Modell Diagnosis and Research Center, Ghent University Hospital, Ghent, Belgium; 4 Department of Biomedical Sciences, Institute of Tropical Medicine and University of Antwerp, Antwerp, Belgium

**Keywords:** Saline, Sodium chloride, SARS-CoV-2, COVID-19, Mucociliary clearance, Acute respiratory distress syndrome

## Abstract

**Purpose:**

Nasal irrigation or nebulizing aerosol of isotonic or hypertonic saline is a traditional method for respiratory or nasal care. A recent small study in outpatients with COVID-19 without acute respiratory distress syndrome suggests substantial symptom resolution. We therefore analyzed pharmacological/pharmacodynamic effects of isotonic or hypertonic saline, relevant to SARS-CoV-2 infection and respiratory care.

**Methods:**

Mixed search method.

**Results:**

Due to its wetting properties, saline achieves an improved spreading of alveolar lining fluid and has been shown to reduce bio-aerosols and viral load. Saline provides moisture to respiratory epithelia and gels mucus, promotes ciliary beating, and improves mucociliary clearance. Coronaviruses and SARS-CoV-2 damage ciliated epithelium in the nose and airways. Saline inhibits SARS-CoV-2 replication in Vero cells; possible interactions involve the viral ACE2-entry mechanism (chloride-dependent ACE2 configuration), furin and 3CLpro (inhibition by NaCl), and the sodium channel ENaC. Saline shifts myeloperoxidase activity in epithelial or phagocytic cells to produce hypochlorous acid. Clinically, nasal or respiratory airway care with saline reduces symptoms of seasonal coronaviruses and other common cold viruses. Its use as aerosol reduces hospitalization rates for bronchiolitis in children. Preliminary data suggest symptom reduction in symptomatic COVID-19 patients if saline is initiated within 48 h of symptom onset.

**Conclusions:**

Saline interacts at various levels relevant to nasal or respiratory hygiene (nasal irrigation, gargling or aerosol). If used from the onset of common cold symptoms, it may represent a useful add-on to first-line interventions for COVID-19. Formal evaluation in mild COVID-19 is desirable as to establish efficacy and optimal treatment regimens.

**Supplementary Information:**

The online version contains supplementary material available at 10.1007/s00228-021-03102-3.

## Introduction

The use of “Atemwegpflege” (care of the airways) or nasal care is a traditional German practice, as to provide moist to the airways and can be achieved by nasal sprays or inhalation of nebulized isotonic or hypertonic saline (Kochsalzlösung) [1–8]. This practice is being promoted by lung specialists, health care, and consumer organizations during COVID-19, whereby the use of nebulizing/aerosols with saline is recommended, either stating that, while it may not change the risk of infection, it helps to mitigate the first symptoms or claiming that it effectively can “dam” (thus reduce) virus infection (“Einfaches Inhalieren kann Tröpfcheninfektion effektiv eindämmern”) [2–6]. Recently, also the Deutsche Gesellschaft für Krankenhaushygiene (DGKH) has formulated gargling/rinse measures to contain SARS-CoV-2 transmission in household, nursing homes, and schools [9].

Whether nebulizing isotonic or hypertonic saline may help to alleviate shortage of breath is not known. Respiratory secretions due to COVID-19 infection may behave similarly as those of a severe bronchitis or bronchiolitis; cough can be dry, but secretions can be clear to mucopurulent, and thus need to be mobilized to be removed from the airways. Aerosol use was actively discouraged in Belgium, as it is believed to create more risks for viral transmission, according to the APB (official pharmacist organization in Belgium) and Sciensano reports [10–12]. Also the World Health Organization (WHO) discourages the use of aerosolizing procedures in general [13]. This risk was also raised at some stage in Germany, because of the fear that this technique would generate bio-aerosol drops, which could promote virus spread. This concept however was contradicted by a positioning statement of the German pneumologists [14]. A recent small study in outpatients with COVID-19 without acute respiratory distress syndrome (ARDS) suggests substantial symptom resolution with hypertonic saline [15, 16].

We therefore investigated the mechanisms by which saline nasal spray/irrigation or aerosol may limit SARS-CoV-2 infectivity and spread. In particular, we discuss the evidence from the literature about the effects of saline on bio-aerosol formation, mucus, alveolar lining fluid (ALF), ciliary beat and mucociliary clearance (MCC), the angiotensin-converting enzyme 2 (ACE2), the sodium channel (ENaC), viral replication (host protease furin, viral protein 3CL^pro^), and formation of hypochlorous acid (HOCl), for their interaction with SARS-CoV-2. The assessment is not meant to address a role of saline in severe COVID-19 ARDS. We also briefly reviewed relevant clinical data and formulate recommendations in support of isotonic or hypertonic saline as a simple rinse or aerosol for an early reassuring intervention for upper respiratory infection and COVID-19.

## Methods

A mixed method approach was taken. At first, information on epidemiological data and treatments of COVID-19 was searched for on the local Internet, as available from the official national sites and as promoted by respectable health care-related organizations in German and Dutch to consumers. As this analysis revealed a common recommendation of use of saline in the frame of COVID-19 in Germany, first systematic searches on established relevant keywords related to saline were performed on PubMed. This was followed by broader searches on new aspects as research progressed and suggested potential other mechanisms of saline relevant to COVID-19. Publications that focused on chronic respiratory diseases were not retained, unless if relevant to discriminate effects from a pharmacodynamic, pathophysiological, or safety point of view. Similarly, the search for relevant clinical effects of oral, nasal, and respiratory hygiene with saline was limited to keywords related to acute upper respiratory tract infections, with or without COVID-19 (SARS-CoV-2). For the pathways followed and the keywords and restrictions used to handle the vast amount of publications on saline and NaCl on PubMed, see Supplement 1.

To note: in this paper, isotonic saline refers to 0.9% NaCl (also called physiologic serum), while hypertonic saline refers to concentrations of 2% and above (commonly 3–7% in experimental and clinical studies and formulation in the market). “Aerosol” refers to nebulizing iso- or hypertonic saline, using a mist-forming device for inhalation and humidification to clear the airways and to remove phlegm in viral respiratory infections. We use the term “bio-aerosol”, when referring to the micro-droplets, spontaneously produced during exhalation, such as during speaking, singing and coughing.

## Results

**Fig. 1:**
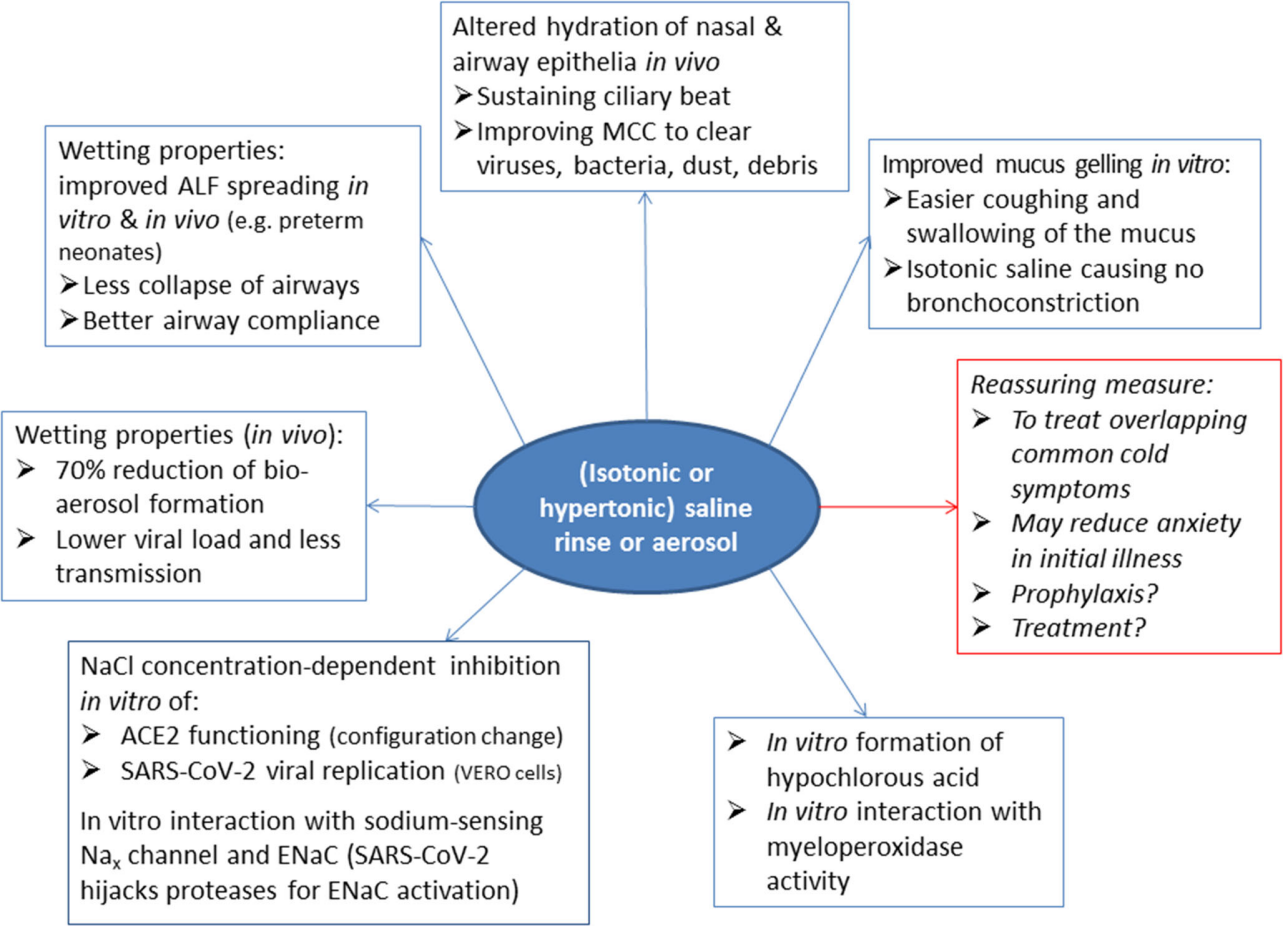
Proposed mechanisms of saline relevant to nasal or respiratory hygiene during COVID-19. Bio-aerosol refers to micro-droplets, spontaneously produced during exhalation, such as during speaking, singing, and coughing; aerosol refers to nebulizing and inhaling saline for inhalation in the respiratory airways, while saline rinse refers to nasal spraying/oral gargling of saline. ALF, alveolar lining fluid; isotonic, 0.9% NaCl (or 9 g/L); and hypertonic, varying concentrations above 0.9% NaCl (2%-7%, often 3%). ACE2 angiotensin-converting enzyme 2; MCC mucociliary clearance

The effects of isotonic and/or hypertonic saline are summarized in Fig. 1. They include the wetting/gelling properties, effect on MCC and hydration, SARS-CoV-2 viral replication and underlying mechanisms, as well as effect on the formation of hypochlorous acid (HOCl).

### Wetting effect on ALF spreading and bio-aerosol formation

Saline changes the physicochemical properties of ALF, mucus, and vesicles/bio-aerosols, thereby affecting the molecular behaviour of ionic and non-ionic surfactants, proteins, and phospholipids in particular. Collectively, these effects are referred to as “wetting properties” of NaCl [17, 18], underlying 2 relevant phenomena: (1) better ALF spreading and (2) suppression of bio-aerosol formation.

1. Surface tension is an important factor in alveolar wetting, the MCC and the phenomenon of capillarity. The alveolar cells produce surfactant that decreases the surface tension in the airways, so reducing the amount of energy required to expand the lungs. From a pathophysiological perspective, the role of pulmonary surfactant in wetting, re-spreading, and compressing the ALF to ultra-low surface tensions is a mechanism that is well-known from preterm children. Such infants might suffer from infant respiratory distress syndrome, characterized by a lack of lung surfactant, which is needed to reduce surface tension forces and is critical for normal lung inflation [19, 20]. Isotonic saline aerosol has been proven to remediate this problem by its wetting properties and to be lifesaving by improving airway compliance [19, 20]. Alveolar type II epithelial cells (AT2) promote the biosynthesis of lung surfactant. SARS-CoV-2 attacks the AT2 cells, causing defective functionality of these cells, which may cause exhaustion of pulmonary surfactant, raise the alveolar surface tension, and finally lead to alveolar collapse [21, 22]. Hence, the wetting properties of NaCl may provide a benefit in reducing surface tensions, thus improving airway compliance.
2. Airway surface liquid (ASL) or saliva droplets carrying the virus are believed to convert into a bio-aerosol infecting the environment and bystanders, and therefore, the use of aerosolizing procedures has been discouraged by several authorities [10–13]. Yet, one should not confound saline aerosol with viral bio-aerosols produced after harvesting from cell cultures, or with bio-aerosol-generating procedures in the hospital (for more information, see Supplement 2): to-date, independent reviews about bio-aerosol generating procedures did not find enhanced risks for transmission of SARS-CoV-2 with nebulizing saline aerosol. In the studies by Edwards [23–25] (Table 1), the delivery of isotonic saline aerosol, nebulized over 6 min, reduced the release of exhaled bio-aerosols from the lungs by an average of 72% (lasting up to 6 hours), an effect persisting over 6 h upon nebulizing 1.29% CaCl_2_ diluted in 0.9% NaCl. The highest effect was obtained in high bio-aerosol emitters [23, 26]. Aerosol administration of saline to the airways particularly diminished the exhalation of smaller particles that facial masks fail to filter out [23]. The effect of 6 min of isotonic saline nebulization on bio-aerosols was furthermore studied in a simulated cough model, producing under bursts of air a bio-aerosol of mucus mimetic. Saline reduced the fine mucin mimetic aerosol, while increased the droplet size of the mucus mimetic (volume-averaged median size 320 nm) instantaneously to 1 μm, and further to 65 μm at 30 min and 30 μM at 60 min [25]. Nebulizing aerosol in a swine model of influenza led to the inhibition of the viral airborne transmission [25]. The setup of another study by Simonds et al., simultaneously using non-invasive ventilation, did however not allow to draw firm conclusions [27]. The German positioning paper on COVID-19 by pneumologists acknowledges the relevance of this property of nebulized isotonic saline, concluding “Although nebulizers with nozzles increase the amount of aerosol in room air, they do not increase the risk of infection for medical staff. The inhalation of isotonic saline solution significantly reduces (bio)aerosol release from the lungs” [14].

**Table 1:** Effects of nebulizing, rinsing and wetting with saline on bio-aerosol formation

Report/study of	Method	Results and (proposed) mechanism
Nebulized (aerosol) in human		
Saline (0.9%) [23, 26]	Assessment of exhaled bio-aerosol particles after saline or a surfactant formulation	– Number of exhaled bio-aerosol particles was reduced by 72% with nebulized saline compared with surfactant, particularly in “high-producers” – Median droplet size containing surfactant was smaller compared with saline – Effect lasting up to 6 h
CaCl_2_ (1.29%) diluted in saline (0.9%) [24]	Assessment of exhaled bio-aerosol particles after saline or a surfactant formulation	– Number of exhaled bio-aerosol particles was reduced as with nebulized saline – Effect lasting beyond 6 h
CaCl_2_ (1.29%-12.9%) diluted in saline (0.9%) [25]	Open-label trial in volunteers and influenza swine model	– Fast production (within 15min) of less and finer bio-aerosol lasting (up to at least 6 h) – Suppression was most pronounced (99%) among those who exhale large numbers of particles – Aerosol administration of these salts to the airways diminishes the exhalation of the small particles that face masks fail to filter – In an influenza swine model, it completely blocked airborne transmission of disease – CaCl_2_ enhanced the effects of saline; the lowest CaCl_2_ concentration was already effective. The effect is ion-dependent ( (not observed by adding MgCl_2_)
Saline (0.9%) [27]	Nebulizer treatment while various aerosol-generating procedures	– Small- and medium-size aerosol/droplet generation, no increase in large-size droplet count – Systematic error possible: not investigated which particles originated from patient or nebulizer, and whether viruses could be isolated from aerosol
Rinse effect in human		
Saline nasal lavage (0.9%) [28]	Viral concentrations after single nasal lavage of infected volunteers	– Lower viral titres after saline nasal lavage in rhinovirus infections; after a single rinse, titres only returned to initial values after day 5
Saline nasal irrigation & gargling (3%) [29]	Open-label, controlled trial in patients with common cold (coronaviruses other than SARS-CoV-2)	– Hypertonic saline reduced transmission within household contacts by 35% (P = 0.006) as compared to controls – 30% more individuals had reduction in viral shedding by ≥0.5 log10 per day
Mechanistic (physicochemical) effects and in vitro studies		
Saline bio-aerosol (0.9%) + mucins [30]	Mechanistic study/biophysical characterization in presence of mucins (confirmed in bull calves)	Charge shielding of mucin or mucin-like macromolecules that consequently undergo gelation, stabilizing ALF/air interface and reducing its breakup, resulting in a reduced tendency of the ALF to disintegrate into very small droplets
Saline droplets (emulsion) [31]	Mechanistic study of NaCl droplets ± surfactant	Added to nanoemulsions, NaCl makes finer micellar droplets “aggregate”, making the droplet size distribution to move to a bigger size range (so will lead to faster deposition), while surfactant in contrast breaks up the droplets to smaller sizes
Saline phospholipid droplets [32]	Mechanistic study of effect of NaCl on phospholipid vesicles and bilayers	– Na^+^ and Cl^−^ binds with the lipid head and induces strong hydrophobic repulsion on the lipid tail – This leads to enhanced hydrophobic repulsion on lipids and so forces lipids to attach firmly on the surface substrate Much larger external energy is needed for vesicle formation in salt solutions than in pure water
Sodium chloride aerosol [35]	Mechanistic study of NaCl and corn oil (bio)aerosol on filtration by face mask materials (FFP1, N95, P100, and elastomeric half-mask respirators)	– NaCl bio-aerosol (even if consisting of finer droplets) penetrates less in face mask material, and thus is better filtered out, as compared with a corn oil bio-aerosol – The generally larger oily droplet aerosol was shown to penetrate deeper in the mask materials So, the ionic nature of NaCl aerosol leads to better capture of bio-aerosol by the mask material
Saline +/- surfactant bio-aerosol (+/- influenza virus particles) [38]	Mechanistic study in evaporated droplets	– Salinity affects the structure of viral particles, whereas processes at the air-liquid interface drive virus inactivation in droplets, also depending on droplet composition and RH – Bio-aerosols of enveloped viruses would fail to undergo rapid rehydration upon entry of the nearly saturated humidity of the respiratory tract, as surfactant arrangement in the droplets would inhibit the reabsorption of water
Saline ± surfactant bio-aerosol (± influenza virus particles) [39]	Study of viral decay in droplets evaporated at different RH and concentrations of saline and protein	– Viability depends on the RH (higher at RH <50% and at 100%) – Viability decreased in saline solutions, the extent dependent on the salt concentrations and presence of protein

*ALF* alveolar lining fluid, *NaCl* saline, sodium chloride, *RH* relative humidity

Table 1 lists furthermore two studies that assessed the effect of nasal rinsing or gargling with saline on viral titres in nasal lavage. These show that saline may also reduce viral transmission via a direct rinse effect of the gargling or nasal rinse with saline [28, 29]: the data support that rinsing leads to considerable direct removal of virus from surface liquid.

From a mechanistic focus, it is proposed that isotonic saline changes the gelling properties of mucin and surface tension of the liquid film on the airway epithelium, leading to less droplet/phospholipid vesicle formation and, as such, to less release of exhaled bio-aerosols [14, 30]. While mucus droplets easily rehydrate after evaporation, their properties are changed by adding NaCl [30]. The mucus is gelled to less and stronger gelled particles, which leads to significantly less droplet formation upon nebulization [30]. This effect is unique to saline and is also observed in presence of other ions that have a more limited effect on aerosol reduction. The effect was also sustained in the presence of surfactants and polysaccharides (dextrans), although the particle count was slightly enhanced by the latter substances: it was moreover found that surface tension was not the determinant factor, but rather the conductivity and viscosity [30]. In the mono-, bi-, or multilayer vesicle technology, saline has been shown to supress the vesicle formation, to induce aggregation of vesicles, and to lead to faster vesicle deposition on surface substrates: the ionic nature makes it more easily to be captured by the substrate layer, while vice versa, as a result, droplets are more difficult to detach from the resulting salt-integrating surface substrate [31, 32]. Saline thus leads to much less bio-aerosol while remaining droplets may average larger sizes, as was shown by Edwards [23–25]: this will affect deposition indirectly in 2 ways: (1) faster deposition of droplets following coughing in the vicinity of the spreader (due to higher gravity and inertia of the larger droplets) [33]; (2) faster deposition of incidentally inhaled droplets in the nose and upper airways, as they are too large to penetrate into the bronchioles and lungs [34]. The bio-aerosol formation for airborne transport will thus become more difficult, as was evidenced by Edwards with saline aerosol in the swine model of influenza [25].

Moreover, not only the droplet size but also the enhanced ionic nature of the bio-aerosol, induced by NaCl wetting, may lead to easier capture /better filtration by filter material. It was shown that a fine NaCl bio-aerosol penetrates less deep in face mask material (FFP1 and N95) as compared with corn oil bio-aerosol [35]. CE-standard criteria for professional face masks ask filter capacities based on testing with plain paraffin aerosol: as surfactants highly change paraffin aerosol properties [36], this may not fully estimate the behaviour of viral-loaded bio-aerosols consisting of enveloped virus or surfactant (phospholipid) vesicles. The fact that the wetting effects of NaCl on bio-aerosol behaviour persist in presence of surfactant [30] may thus be relevant to filtering out viral-loaded bio-aerosol. Salt-covered masks have recently been developed as to destroy more quickly coronavirus droplets when landing on the mask material [37].

At last, saline will also lead to fast shrinkage of droplets upon evaporation in the ambient air (already ongoing at a relative humidity of 90–95%): loss of water induces phase separation, enhanced salinity, and decreased pH in the droplets/particles, as well as re-organization of the phospholipids at the outer air-droplet interface [38, 39]. The hydrophobic nature of the resulting phospholipid-coat was found to impair rehydration of virus-loaded particles [38]. Salt concentrations have been shown to adversely affect the survival of influenza virus in bio-aerosol [39], while increased hydrophobicity of the particles lead to faster and reduced deposition in the airways according to aerosol technology [34].

### Role of saline in the MCC

The MCC is the major primary innate defence mechanism of the nose, airways, and lungs, continuously clearing these from dust, infectious, and other particles by the ciliary movements. Using different techniques, it has been shown that isotonic saline (aerosol or rinse) induces a positive effect on the ciliary beat frequency, reverses ciliostasis and promotes the MCC, both under physiological and damage-induced conditions [40–44]. Factors that play a role include the osmolality of the saline, the hydration of the ALF and the composition of the mucins.

1. *Osmolality of saline*. Pure water severely damages the normal human nasal epithelial cells, while isotonic saline (in contrast to hypo- and hypertonic saline) does not affect their morphology [45, 46]. Hypertonic saline has been shown to decrease the ciliary movement in human nasal epithelium [43, 47], while others report a faster MCC in healthy subjects, after-single dose nasal irrigation, or in the airways at 30 min, but not 4 h after inhalation (attributed to depletion of airway mucin) [44–50]. Hypertonic saline induces osmotic pressure, but has also been found to decrease the potential difference in nasal epithelia—a rapid, reversible, and dose-related effect indicating a direct effect of NaCl on ion transport across the human airway epithelium (not just attributable to a simultaneous change in osmolarity) [51]. Hypertonic saline has also been found to affect the nasal epithelial permeability [52, 53]. Its use may be associated with nasal burning/irritation [54], while both hypo- and hypertonic saline aerosols may induce bronchoconstriction or cough, as observed in patients with asthma or moderate to severe chronic obstructive pulmonary disease [55–58].
2. *Hydration of the ALF*. The MCC requires coordination between the periciliary liquid near the cell surface and the overlaying transported mucus layer [59, 60]. Therefore, these layers need to be appropriately hydrated in the lungs and airways, allowing the cilia to beat properly, to move the mucus and to transport the trapped pathogens and particles. The nasal and respiratory mucin forms a gel layer, serving as a liquid reservoir for the periciliary layer [61]. The height of the ALF or ASL and hydration of the periciliary liquid layer depend on opposing mechanisms in water transport: the outward chloride (Cl^−^) secretory transport through apical chloride channels (CFTR and CACC mediated), and the inward movement of water following active (re)absorption of Na^+^ through apical sodium channels ENaC. These ion transporting actions occur in concertation with the basolateral Na^+^/K^+^-ATPase, located in the ciliated cells [62–64]. Whereas several more ion transport processes are involved, Na^+^ and Cl^−^ are the main drivers of the fluid movements. While the periciliary liquid layer normally contains NaCl in an amount below 50mM (< 0.29%), the upper layer of ASL would contain concentrations of these ions above 100mM: this gradient ensures effective and sustained transepithelial transport of ions and water, as well as effective ciliary beating [64]. Saline thus contributes to the hydration status of the nasal and airway mucosa, while it also regulates the height of the ALF.
3. *Mucus properties.* The properties of the mucins affect the MCC as well. Healthy mucus is a gel with low viscosity and elasticity, easily transported by ciliary action [61]. In contrast, pathologic mucus, such as produced during chronic pulmonary diseases or ARDS, has higher viscosity and elasticity: it is less easily cleared from the airways. Impaired mucus clearance induces cough, airway obstruction, and dyspnoea [61]. In elderly patients in particular, there is often already stasis of thick, dehydrated mucus within the nasal cavities and nasopharynx, leading to postnasal drip, cough, and globus (pharyngeus) sensation [65]. Decreased MCC may lead to persistent accumulation of mucus, so providing an environment for microbial growth of pathogens in the respiratory system and contributing to secondary infections and inflammation [66]. Saline equilibrates in the mucus, which leads to several actions of NaCl on the mucus: better diffusibility of pathogens into the mucin [67]; enhanced entrapment in the gelled mucus (gelling observed at 100 mM (0.6%) NaCl [30, 68]); reduced adhesion of mucins to the epithelium (observed at 0.9% NaCl) [69]; enhanced ciliary transportability and clearance (already observed from 0.5% (90 mM) saline onwards) [61, 70]; and so easier coughing up and swallowing of mucins, better cough clearance, and relief of (chronic) cough [69, 71]. As saline aerosol helps to fluidize the mucins in the deeper airway layers, also mucin-attached pathogens involved in secondary pulmonary infections are more easily cleared [66]. While most of these effects are obtained by isotonic or lower NaCl concentrations, highly dehydrated sputum and ASL, such as occurring in cystic fibrosis and chronic bronchopulmonary disease, require higher (hypertonic) saline concentrations, as to equilibrate the sputum for maximal transport by drawing additional water onto the ASL [71–73].

Saline thus contributes by different mechanisms to optimizing the MCC and to protect the airways against infection. This has been nicely illustrated in porcine ciliated airways: the viral yield of Influenza A virus in the ciliated epithelium was about two- to threefold higher 24 to 48-h post-infection in the case of ciliostasis, as compared to normal ciliary activity, while saline (up to 2%) led to full recovery of the ciliary activity and reversal of ciliostasis, thereby impeding the viral infection [42]; however, at NaCl concentration exceeding 2%, the recovery rate decreased the more the saline concentrations increased up to 11% NaCl hypertonicity [42]. This further underlines the modulatory role of the salt concentration.

Noteworthy, additional factors might affect the MCC. Adding bronchodilators to saline aerosols, such as salbutamol or terbutaline, may improve the nasal MCC [74, 75]. In contrast, many preservatives, excipients, antimicrobial agents, lidocaine/ anaesthetics, opioids, and mucolytics, such as acetyl cysteine, decrease the MCC [74, 76].

The ciliary activity and MCC are furthermore influenced by body and ambient temperature, pH, and moisture [62, 77]. These factors also modulate the dryness of the oronasal mucosa.

### Role of saline in mucosal hydration

The nasal cavity gets moistened as warm breath condensate, originating from the lungs, moves over the cooler nasal epithelial surface during expiration. Drying of the respiratory mucus and excessive dryness in the nose are common problems that have been associated with reduced ciliary function and decreased MCC [41, 77]. For instance, passage of dry cold air current or chilling depresses the mucous membrane temperature and ciliary movement, which manifests to a greater extent in the nasopharynx than in postnasal spaces [77]. The speed of warming up of inhaled air depends on the respiratory frequency and volume of air inhaled, but hyperventilation leads to faster drying of the mucosa and reduced clearance from the lungs [41]. Elderly people may more frequently suffer from a dehydrated mucosa, while they also show altered cilia, slower ciliary beating, changed properties of mucus and slower MCC [65, 78]. Nasal dryness is also affected by the body temperature, the temperature of the nose fluctuating directly with the core body temperature [77]. Hence, also during high fever, there may be faster drying of the respiratory mucosa, making elderly patients with fever even more susceptible to viral aggression. Additionally, the prolonged use of facial masks is associated with dehydrated mucosa of the oronasal cavity [79]. This is not surprising, as the conditioning mechanism, consisting of mucosal cooling during inhalation and condensation of the saturated, warm, exhaled air [77, 80], is drastically reduced.

As a consequence, humidification with saline aerosol may be beneficial. It is not known whether the hydration by saline occurs via promoting or restoring the ciliary function and/or other factors. It has been shown that “a fringe of ciliary activity persists” as long as there is sufficient moisture [77]. However, if dryness of nasal epithelium lasted longer than 15–18 min in vitro, air humidification or water flushing of the epithelium could no longer restore the ciliary movement, while only isotonic saline or Ringer’s solutions did so [77]. In line with these observations on ciliary movement, the process of mucosal hydration in surface airway epithelia was found not to be dependent on the cilia itself, but in the first place on the presence of soluble mediators in the ASL [81]. The hydrating effects of saline (0.9–7.0%) have been well documented in chronic respiratory diseases that are associated with dehydrated mucins and reduced ASL in airways and/or lungs [72, 73, 82]. Yet, for a dry nose or larynx, isotonic saline is the concentration of choice to moisten the dry nasal mucosa [83, 84]. Health care professionals wearing well-fitting face masks report to suffer from less dryness in the nose and mouth if using isotonic saline nasal rinse/spray twice daily (morning/evening) [85]. Mucosal hydration by saline may also play a role in dry cough: nasal isotonic saline has been found to reduce dry cough associated with acute respiratory illness, such as during flu or common cold [86].

### Interactions of saline with SARS-CoV 2]

NaCl can directly affect SARS-CoV-2 virus infectivity by interacting with its ionic or electrostatic charges. NaCl is listed as an antimicrobial against coronaviruses MHV-2, MHV-N (mouse hepatitis viruses), and CCV (canine coronavirus), as these viruses lose infectivity after exposure to NaCl 0.23% [87]. Machado et al. showed that the SARS-CoV-2 replication is dose-dependently inhibited by saline (0.8–1.7% NaCl) in Vero CL-81 cells [88]. Inhibition of viral replication started from 0.6% onwards, increasing to 50% inhibition at 0.9% (isotonic) saline and reaching 100% at 1.5% (so mildly hypertonic) saline. Saline, however, had no direct effect on the SARS-CoV-2 itself: saline-pretreatment of the virus could not inhibit subsequent viral replication in the host cells. The authors proposed different underlying mechanisms: (1) NaCl-induced hyperosmotic stress leading to the SARS-CoV-2 inhibition (yet, unlikely, as no direct effect on the virus was shown), (2) decreased expression of the phosphokinase C signalling pathway (yet, this would require time for down-regulation), and (3) depolarization of the host cells via ENaC and its sodium sensor, the Na_x_ channel, thereby over-stimulating ENaC and leading to electrolyte movements stressing the mitochondria (unlikely as rather ENaC dysregulation occurs—see below).

We identified five interactions of saline, relevant to viral tropism, that have been documented and thus are reachable, already at concentrations with isotonic saline:

1. *Chloride- and pH-sensitive conformation of ACE2.* ACE2 is the entry receptor of SARS-CoV-2 and is present in the nose, oropharynx, and airways (particularly in ciliated cells) [89–91]: increasing saline concentrations have been shown to induce dose-dependently immediate steric hindrance in the ACE-2 receptor configuration for binding of angiotensin (Ang) II: the inhibition starts at 100 mM (0.58%) NaCl which is close to a minimal effective inhibitory concentration of saline on SARS-CoV-2 replication (0.8% NaCl) [88, 92, 93]. Also relevant pH-mediated effects on binding of the endosomal ACE2 receptor have been reported for the first SARS-CoV virus: these effects would occur through interaction at a terminal glycosylation site, thereby inducing less virus-receptor binding [94].
2. *Inhibition of the viral protease 3CLpro*. Human coronaviruses, such as SARS-CoV and SARS-CoV-2, typically harbour and make use of 3CLpro, a chymotrypsin-like cysteine protease that regulates the viral replication machinery. There is no significant blocking effect up to 50 mM of NaCl on 3CLpro. However, higher concentrations (100 mM (~0.6%)) of NaCl does exhibit an almost full 3CLpro block, while the enzyme activity is also strongly pH dependent (fully blocked at pH<6.0) [95–99]. Significant inhibition and disruptive effects on the enzyme’s dimerization occur as NaCl concentrations move to values of >100 mM [96, 100, 101].
3. *Inhibition of the host protease furin*. TMPRSS2 and furin are involved in the proteolytic activation (priming) of SARS-CoV-2 spike protein [102–104]. The proteolytic activity of furin was previously found to be inhibited by NaCl, starting at 75 mM (~0.4%) onwards, and achieving > 90% inhibition at 100 mM (0.6%), and complete inhibition at 200 mM (~1.2%). No inhibitory effect was observed with KCl or CaCl_2_ solutions that rather stimulated furin [105]. The inhibition is concordant with the finding of a furin insertion site by Anand et al that seems to be uniquely acquired by SARS-CoV-2 [112].
4. *PH-shifts in nasal and airway environment*. The pH of the incubation medium directly affects the unfolding of the SARS-CoV-2 spikes, which is hampered at pH 4.5 [106]. A role of mild acidification in impeding SARS-CoV-2 replication is also suggested by the outcomes of in vitro studies in Vero-cells: complete inhibition of viral replication was observed with pure (unbuffered) saline (pH 5.5) and with NH_4_Cl (pH range 4.6–6.0), but not with phosphate buffered saline (PBS pH 7.4) [88, 107]. Both the pH and Na-concentration might be relevant to viral replications, similarly as suggested by the volume effects of added unbuffered saline, various sodium-containing buffers, and different pH levels as transport medium, observed with external polymerase on the detection levels of extraction-free SARS-CoV-2 RT-PCR of saliva samples [108]. The pH of the nasal or airway surface liquid may be relevant to contracting the virus, the more as the healthy nose and airways, as well as sputum, have a slightly acidic pH (pH 5.5–6.5): this pH changes to more alkaline pH (pH 7.2–8.3) during common colds and chronic respiratory conditions [109]. The mechanisms whereby saline acts acidic in vivo are complex and involve various channels in the respiratory epithelium [109, 110].
5. *Interaction with ENaC*. As already discussed, ENaC is the main mechanism for maintaining the height of ASL and regulating the ALF by stimulating the sodium absorption, so also preventing immersion and immobilization of the ciliated epithelium [62–64, 111]. Based on protein sequencing, Anand et al. [112] identified a S1/S2 cleavage site in SARS-CoV-2 that can mimic the proteolytic activation of human ENaC: the virus can hijack several proteases that are involved in the activation and regulation of ENaC (TMPRRSS2, furin, prostasin, and matriptase [113–115]), furin in particular in view of a unique furin insertion site in its S protein [112]. Hence, this may lead to dysregulation of ENaC and the fluid balance in the lungs [116]. Yet, fluid homeostasis is also regulated by the cooperation between ENaC and the sodium sensing Na_x_ channel, directly activating ENaC [117, 118]. This mobilization and activation of ENaC via the Na_x_ sensing channel activating ENaC is likely to take place with saline because the threshold for Na_x_ activation in vitro is 150 mM (0.88%) extracellular Na^+^ [117] and thus reachable with isotonic saline. In line with this mechanism, it was shown that iso- and hypertonic saline administered to an in vivo rat model was able to overcome pharmacological ENaC blockade of lung, leading to reabsorption of lung secretions [119].

### Myeloperoxidase (MPO) activity

The interactions between NaCl and MPO are very complex, while those observed between SARS-CoV-2 and MPO so far have not been elucidated. The main actions of saline on MPO are discussed in Supplement 3. Basically, addition of NaCl to H_2_O_2_ producing cells in vitro will shift the MPO activity from peroxidation (to form H_2_O_2_) towards chlorination (to form HOCl). NaCl thus leads to the production of increased virucidal HOCl (bleach): this effect on MPO is already observed at 10 mM NaCl (0.058%), while the virucidal action of HOCl is observed at 0.09–1.7% NaCl [120, 121]. Moreover, whereas human MPO activity in neutrophils is active from 25 to 140 mM NaCl (0.14–0.82%) [122], the phagocytosis of pathogens requires continuous supply of chloride (Cl^−^) to sustain the HOCl generation in the phagosomes [123]. Alternatively, because Cl^−^ also competes with thiocyanate as a natural substrate for MPO activity, saline may shift the substrate thiocyanate towards antioxidant pathways, while its concomitant presence with thiocyanate increases the yield of virucidal hypothiocyanite formation [124]. NaCl has also been shown to interact with reactive oxygen species production, as well as with extracellular neutrophil trap formation [125]. These complex interactions are beyond the scope of this review but have received substantial attention in the context of (severe) COVID-19 [see Supplement 3]. The effects of NaCl on MPO may be clinically relevant in less severe presentations as well, as a pilot study using aerosols of electrolyzed saline (so containing HOCl) proved to be highly effective in the treatment and viral clearance of SARS-CoV-2 in ambulatory patients with COVID-19 [126].

### Translation of the mechanisms into clinical benefits

Supplement 4 reviews the most relevant sources supporting the use of isotonic and hypertonic saline aerosol in (non-COVID-19) ARDS and bronchiolitis, as well the scarce data obtained so far in COVID-19 positive patients. In ARDS or bronchiolitis by other respiratory agents, such as the syncytial respiratory virus infection, aerosols of both isotonic and hypertonic saline have been shown to exert beneficial effects, such as improving respiration and the clinical severity scores, while hospitalization rate and/or duration were reduced, in particular in children (Table 2A, Supplement 4). Yet, overall, better outcomes were seen with isotonic as compared to hypertonic saline, in part because the latter was associated with worse cough in infants. In order to treat and prevent common cold and/or flu, studies support both acute and preventive use of saline irrigation (Table 2B, Supplement 4). The benefit of saline irrigation to promote recovery from common cold is acknowledged by the WHO [127, 128]. With regard to treatment of COVID-19 patients, limited pilot studies are available thus far. Two studies (one in upper respiratory tract infection caused by seasonal coronaviruses or common cold virus [29], one in symptomatic COVID-19 [15]) use a control group and support the early use of nasal and oral rinse with hypertonic saline, started within 48 h of symptom onset [15, 29]. Two observational studies are relevant from a safety perspective. A survey of COVID-19 patients with anosmia mentioned the use of saline by 57% of the patients: anosmia resolved over time but the recovery was not documented by initial treatment; to note is that none was hospitalized or developed ARDS [129]. In a German study in patients with a more severe spectrum of illness, daily isotonic saline aerosol was used by 60 patients with COVID-19 ARDS. Administration was started prior to (bio-aerosol generating) non-invasive ventilation procedures: only 3 deteriorated and required intubation (all with severe comorbidity, of which one died); no health care workers got infected [130]. For more details, we refer to Supplement 4.

**Table 2:** (A) Pharmacological effects of saline in the setting of COVID-19 and effective concentrations. (B) Reference values of Na^+^ and Cl^-^ in nose, airway and lung fluids

Parameter	Physiology/pathophysiology relevant to common cold/respiratory infection	Pathophysiology with regard to SARS-CoV-2	Pharmacological effects of saline or reference concentrations
(A) Pharmacological effects of saline			
Wetting—effect on ALF			
Bio-aerosol/vesicle generation and ALF spreading	Airborne transmission of COVID-19 by aerosol such as speaking Superspreading events in closed and non-ventilated areas [10–13, 130–132]	SARS-CoV-2 attacks surfactant producing alveolar type II epithelial cells (AT2), raise the alveolar surface tension and so lead to alveolar collapse [21, 22] Infection of lower airways and lungs thought to occur via micro-aspiration of ultrafine droplets [144]	NaCl (0.9%) leads to: - Wetting and fluid aggregation, leading to: - suppression of aerosol/vesicle formation: [72% reduction of bio-aerosol formation after 6 min aerosol, lasting up to 6 h] - Easier deposition/filtering of heavier /larger drops—so less easily deposition in the deeper airways and lungs - Reduction of viral shedding (shown for other viruses) - Improved ALF spreading and airway compliance (applied in premature newborns) [17–35]
Viral shedding of rhinovirus/coronavirus	Transmission of common cold viruses and COVID-19 occurs by apical shedding of viral particles and/or exosomes (secretion of virus containing vesicles) [134–136]	Viral shedding of SARS-CoV-2 may take place during infection [134–136]	NaCl (0.9%-3%) provides (shown for viruses other than SARS-CoV-2): - *In vivo* rinse effect, causing lower viral titres (5 days until back to initial values) - *In vivo* reduces viral shedding as demonstrated for other viruses (rhino- and other coronaviruses) [28, 29]
Mucociliary clearance (MCC)			
MCC (ciliary beat)	Primary defence mechanism to expel pathogens, viral particles and debris	SARS-CoV-2 preferentially targets ciliated cells in nose, nasopharynx, airways, olfactory bulb and reduces the MCC [134–136]	NaCl (0.9%) promotes ciliary beat and MCC *in vitro* and *in vivo* Dependent on osmolality/ionic strength - is reduced with hypotonic saline - variable effect with hypertonic saline in vitro: effects observed on the epithelial cell membrane (altered electrical conductance, permeability, cell deformation) [40–58]
Mucins, containing sialic acids	Captures pathogens, viral particles and debris to remove these by MCC or upon coughing	SARS-CoV-2 spike protein binds sialic acid (if not buffered at pH>7) and is found in sputum [137, 145]	NaCl gels the mucus *in vitro* [shown at >90 mM—0.6%], so altering the MCC and cough clearance [> 0.9%] [30, 67, 68]
Cough clearance	Associated with MCC Stasis of thick, dehydrated mucus within the nasal cavities and nasopharynx lead to postnasal drip and cough	SARS-CoV-2 is frequently associated with cough, while present in sputum [137]	NaCl (0.6%) gels the sputum and (0.9%) reduces its adhesion, so promoting cough clearance [69–71]
Mucosal hydration			
Mucosal (de)hydration	Periciliary fluid normally contains <50 mM NaCl (0.29%), while higher concentrations at the tips (>100 mM – 137 mM) as to maintain absorption gradient and ciliary movement. Ciliary beat and MCC is inhibited by dry mucosa.	SARS-CoV-2 may lead to dysregulation of Na^+^-homeostasis in the airways/lung [116] More severe SARS-CoV-2 is frequently associated with conditions of dry mucosa, such as in the elderly, or during wearing face masks, fever, and infection [65, 78, 79, 153]	In vitro and in vivo - Isotonic (0.9% NaCl): hydrating effect paralleling ciliary functioning - Hypertonic (usually 3% NaCl): hydrates and has strong rinsing effect (osmosis); [59–65, 77, 79–84]
Viral replication			
Viral replication	Replication of SARS-CoV-2 virus leads to overlapping common cold symptoms	*In vitro* replication of the virus is inhibited by pure saline [88] The effect is pH and Na- dependent [106–108]	NaCl inhibits SARS-CoV-2 replication in vitro in Vero-cells: pure saline • MIC: 0.6% • IC_50_: 0.9%—IC_100_: 1.5% [88]
(Na)Cl concentration at ACE2- expressing cells	Strong Cl^-^-dependency of ACE2-receptor : from a certain concentration onwards, NaCl induces steric hindrance of ACE2 receptor for its substrates [92]	ACE2 is the entry receptor for SARS-COV-2 [89–91]	NaCl causing steric hindrance of ACE2 receptor *in vitro*: in HEPES • MIC: 0.5% • IC_50_: 1.4% [92–94]
SARS-CoV-2 protease 3CLpro	3CLpro is a protease shared by coronaviruses: its use upon proteolytic processing of their replicase polyprotein leads to production of infectious virus [95, 96]	SARS-COV-2 uses its own protease 3CLpro for replication [95, 96]	NaCl inhibits SARS-CoV-2 protease 3CLpro (deduced from graph): • MIC_80_: ~0.9% (150 mM) • IC_90_: ~1.8% (300 mM) [95–101]
Host protease furin	Furin contains a sodium-sensor and is inhibited from a certain concentration by NaCl, but not by calcium or potassium chloride [105]	SARS-COV-2 needs TMPRSS2 and has a furin-binding domain to prime the spike forACE2 entry [102–104]	NaCl inhibits furin (deduced from graph): • MIC: ~0.45% (75 mM) • IC_~90%_: ~0.9% (150 mM) • IC_100_: ~1.2% (200 mM) [105]
ENaC	ENaC determines hydration and height of ALF and MCC, as well as drives reabsorption of diluted ALF hypersecretion; ENaC activity is regulated by various proteases and by the sodium sensor Na_x_ [62–64, 111, 117, 118]	SARS-CoV-2 possesses unique furin-insertion site, as well hijacks, so shares several proteases that inhibit/regulate ENaC, leading to less availability for ENaC and so to less fluid absorption in the lungs [112–116]	Na_x_ sensing of NaCl at 0.9%NaCl, stimulating ENaC and so sodium (re)absorption, contributing to the volume control and Na^+^-homeostasis, the control of ALF height and MCC [Threshold Na_x_: ~150 mM] [117] NaCl (0.9–1.5%) allows to overcome secretion in the lungs following pharmacologically induced blockade of ENaC [119]
Myeloperoxidase formation (MPO)			
Hypochlorous acid (HOCl), myeloperoxidase (MPO)	– Antiviral effects of NaCl are attributed to the production of HOCl from Cl- ions – HOCl is mainly produced by MPO : HOCl generation in the phagosomes requires a continuous supply of chloride [120–123]	SARS-CoV-2 is sensitive in vitro to HOCl [120, 121]	NaCl 15-300 mM (= 0.09 to 1.7%) results in HOCl production [120, 121] MPO activity in neutrophil phagosomes increases with increasing NaCl concentrations from 25 mM (0.14%) NaCl onwards [112]
Relevance not found related to SARS-CoV-2			
CFTR	Apical Cl^-^ secretion is mediated mainly by CFTR, relevant to dehydrated ALF (as in CF)	Not reviewed – No specific interactions relevant to viral infection known so far	(?) Rationale remains unclear besides the known effect of hypertonic saline in osmosis creation [so far not found to be relevant to SARS-CoV-2; relevant to conditions characterized by dehydrated ALF, thick mucus and impaired MCC.]
(B) Reference values in body fluids			
Body fluid			Reference concentration of Na^+^ and Cl^-^
Saliva			Low to highly variable :3–125 mM, average 5.6 mM [143]
Nose			
Nasal fluid			Averages: Na^+^: 128–150 mM; Cl^−:^>139 mM [139–141] Highly variable -> range : 85–225 mM Na^+^ [139]
Inspiring via mouth versus via nose			Na^+^:120 mM (mouth), 189 mM (nose) Cl^−^:134 mM (mouth), 205 mM (nose) [139]
Nasal mucus			Na^+^:139–150 mM Cl^−^: 139 mM [139]
Common cold			Na^+^: average 135 mM vs – 185 mM (spontaneous respiration healthy) – 128 mM on sneezing [139]
Airways			
ASL			> 50 mM Na^+^ Cl^-^
Periciliary liquid			≤ 50 mM NaCl [142]
Outer ASL			>100; average 137 mM NaCl [142] + NaCl 0.9%: ALF remains isotonic during the 5 hours assessed ; only hypotonic solutions are not sustained] [142]
Distal airway fluid			122 ± 2 mM Na^+^, 123 ± 4 mM Cl^−^ [144]
Lung			
ALF			82-91 mM for Na^+^ and 82–108 mM for Cl^−^ [141]
Sputum (mixture of saliva and mucus coughed up during infection/disease)			0.74 % (123 mM) NaCl [140]

*ALF* alveolar lining fluid, *CFTR* cystic fibrosis transmembrane conductance regulator, *Cl*^−^ chloride^−^ *HOCl* hypochlorous acid, *IC*_*50*_ inhibitory concentration to inhibit 50%, *IC*_*100*_ inhibitory concentration to inhibit 100%;*MCC* mucociliary clearance, *MIC* minimum inhibitory concentration, *MPO* myeloperoxidase, *Na*_*x*_ sodium channel x, *Na*^+^ sodium

## Discussion

In a majority of qPCR-positive persons, SARS-CoV-2 infection presents as asymptomatic or mild disease; yet, a minority of mainly older persons or patients with comorbidities or immune deficiency will develop severe ARDS. As COVID-19 ARDS is a serious complication, it requires early recognition and comprehensive management. Any preventive or acute treatment proposed for common cold—overlapping with COVID-19—should guarantee sufficient safety, if applied in an early stage. Since transmission may take place through bio-aerosol in the period prior to getting seriously symptomatic, effective hygiene measures, such as gargling or rinses, should be initiated as early as possible, as proposed by the DGKH. Treatment ideally should also be easy, safe, and at low cost. This analysis shows that saline rinse and aerosol are a safe and effective approach to treat and prevent upper respiratory tract infections and common colds, while the evidence presented in this manuscript also provides rational arguments for its application to contain and relieve mild COVID-19 infection, if started early within 48 h after the onset of symptoms. Although formal studies are welcome to address the optimal saline concentration(s) and frequency of applications, the data support the use of both isotonic and hypertonic saline, although for maintenance in the nose and/or lungs isotonic saline may be associated with less adverse events. Our recommendations based on this analysis are summarized in Table 3. These are in line with online recommendations to German consumers [1–7], and provide strong support for the gargling/rinse recommendations by the DGKH for use in the household, nursing homes, and school setting [9]. Our analysis thereby substantiates several mechanisms, relevant to SARS-CoV-2 infection.

**Table 3:** Pharmacy practice recommendations for saline use, based on this literature analysis

• Saline does not destroy SARS-CoV-2 and is thus only to be used as an add-on to basic hygiene measures	
• In case of acute common cold or upper respiratory symptoms in times of COVID-19	
Nasal rinse	From first symptoms of common cold or upper respiratory symptoms Rinse with pure “isotonic” saline (0.9%) 2–3 times/day* • This concentration combines a reliable positive effect on the MCC with desirable partial receptor block of the entry receptor and is well established for treatment or prevention of common cold and upper respiratory symptoms, and as nebulization/aerosol for treatment of bronchiolitis • Isotonic saline is devoid of the side effects that have occasionally been reported for hypertonic saline (effect on cell morphology, increased nasal epithelial permeability, nasal burning/irritation, and when nebulizing: induction of bronchoconstriction or cough) • [*Isotonic saline for nasal rinse unless if hypertonic saline is already used in the frame of other indications, in which case it can be continued] • Heating the saline is not needed; concentrations of salt reached upon inhaling sea salt solution are unknown • No special devices are needed
+ Gargling	In case of common cold symptoms with throat involvement: *Or* in case of COVID-19 positive testing -> Gargling can be done with self-made hypertonic saline 1 table spoon or 20g kitchen salt per litre, or as formulated by DGKH [9]: 1 flat-filled teaspoon in 100 mL: up to 12 times per day -> Don’t swallow; discard in sink
Respiratory care	When using a saline aerosol with a nebulizing apparatus to reduce bio-aerosol formation and/or remove phlegm, hydrate the airways and/or reduce cough -> Continue with habitual strengths of sterile saline concentration (0.9%), unless otherwise indicated, or ask pharmacist -> Follow the cleaning instructions of the manufacturer for the cleaning of the device, to end with hand hygiene -> Preferentially to be performed in well-aerated place or outside
	See your doctor if not getting better and/or if feeling short of breath or/and very sick with high temperature
• Preventive use	
	Daily nasal and oral gargling may be useful in situations, such as also formulated by the DGKH [9]: e.g. • As nasal saline spray for hydration of the nasal mucosa (e.g. when feeling dehydrated due to carrying a mouth mask) • As gargling and nasal rinse, before seeing a frail or immune compromised person, before meals and qPCR-positive household, upon outbreak of respiratory illness in schools, or after visiting an unexpectedly crowded location, and thus if risk of contamination is believed to be higher Distancing and hygiene measures will prevail at any time
• Protective measures: always combine with protective measures	
	Nose and Mouth • Collect superfluous liquid with tissue paper and discard or in water with detergent and safely dispose in sink • Wash and/or safely collect the saline recipients, to end with hand hygiene Nebulization/aerosol • If there is no way to isolate or to aerate the room, use cotton sheets to cover your lap (plus head) to prevent aerosol dispersion • Ventilate the room and wash hands

On one hand, saline provides advantages for preventive uses as a nasal spray or a rinse for the nose and oral cavity, because saliva has been implicated in transmission, while the unique physicochemical properties of saline lead to suppression of droplet formation [23–26]. If administered as a 0.9% saline aerosol for 6 min, the latter effect is the highest among superspreaders and leads to larger droplets that are more easily filtered by facial masks [23, 26]. This mechanism of action of saline was also shown to work experimentally in a swine model by preventing the airborne transmission of flu virus [25]. Its aerosol use, add-on to the strong protective hygiene measures in the hospital during non-invasive ventilation in COVID-19-ARDS patients, was not associated with enhanced transmission [130]. The findings are encouraging and invite to follow the recent recommendation of the DGKH to use gargling and nasal rinse with saline in a number of preventive situations, such as in care setting before meals or activities involving seniors or revalidating persons, family or professional reunions, church or religious gatherings (even if COVID-proof), or for kids in schools [9]. Infected bio-aerosols are thought to be formed during coughing, or also during speaking or singing when bronchial secretions move over the vocal cords [131, 132], while bio-aerosols are insufficiently blocked by cotton and medical masks (even surgical or N95 and if well-fitting) [133]. The unique wetting properties of NaCl identified in this study justify oronasal hygiene to be combined with the current face mask and distancing measures. In particular, maintenance of oronasal hygiene with saline (as aerosol, spray, or rinse) may achieve less penetration of the bio-aerosol through a mask [35]. The finding that upon evaporation the enhanced salinity may deform virus-containing droplets [38] deserves further study, as to find out more on SARS-CoV-2 decay in NaCl/virus-loaded bio-aerosols.

On the other hand, many mechanisms of saline were identified that strengthen the innate primary defence mechanisms, such as promoting the MCC and interaction with MPO. These are complemented by various mechanisms inhibiting SARS-CoV-2 replication—all presenting at the saline concentrations currently in use (Table 2). Multiple mechanisms of action justify early use from the first onset of symptoms of COVID-19. Firstly, these mechanisms of NaCl may prevent massive viral replication and destruction of the cilia in the oral and nasal cavities—possibly by inhibiting the protease activity of furin and 3CLpro: SARS-CoV-2 targets ciliated cells in the nose and airways, releasing virus or abundant secretory vesicles, while also impairing the MCC [134–136]. The SARS-COV-2 spike protein also contains glycosides that effectively bind sialic acids (the main constituents of mucus) [137]: as such, salt-induced gelling of mucus by the slightly acidic saline will improve viral clearance. As evidenced by Table 2, most in vitro pharmacological/pharmacodynamic effects of saline were achievable at concentrations reached by isotonic saline. This is reassuring from a pathophysiological point of view, since SARS-COV-2 mainly initiates infection in the nose and seeds by micro-aspiration to the lower airways, after which tracheal-produced virus would further seed via aspiration into the deep lung [136, 138]. Moreover, the reference values of Na^+^ and Cl^−^ in the relevant fluids (saliva, nasal mucus and fluid (during common cold), periciliary liquid, ASL, ASL and sputum [139–144]) revealed to be generally lower than those in the mM NaCl concentration provided by isotonic saline (see Table 2B). As large volumes of saline can be safely repetitively used, this thus allows achieving relevant concentrations throughout the day to reach the described effects. Also safe long-term use is well documented in chronic lung disease. The analysis further suggests that due to its isotonic nature, saline may also help to reverse the bronchoconstriction in the case of hyper-secreted, thus potentially hypotonic ALF, as well as enhance cough clearance [17–20, 23, 30]. In view of the vast literature of saline, a limitation of this analysis though is that not all effects of saline or mechanisms relevant to SARS-CoV-2 may have been addressed.

To note, nasal and airway pH may be important in contracting COVID-19, in view of the observed pH-effects on protease and viral replication inhibition (Results Part 3). In addition, the pH affects the mucin binding by SARS-CoV-2, which was found to be absent if the mucins were buffered at pH 7.0 [145]. Our recommendation is therefore to use pure saline (pH 5.5) for respiratory hygiene rather than buffered (seawater) sprays, although these may already work to some extent through the simple rinsing effect [86, 146]. Although often lukewarm or heated saline solutions are recommended for rinsing or inhaling, there is no evidence that this leads to better action of salt than the then the use of saline at room temperature [147]. Other nasal spray compositions—with more or buffering ions, surfactants, emulsifiers, excipients, preservatives and/or active substances— may not necessarily lead to the same effects as identified in this analysis for pure saline, or may inhibit the ciliary beating [74–76]. Addition of certain surfactants to liquid formulations may enhance bio-aerosol formation [23, 30, 31] or be ciliotoxic (e.g. polyvidone-iodine [148]), while many (such as virucidal essential oils) may not respect the natural microbiome. Also the DGKH recommends saline formulations without additives, such as preservatives or decongestants [9]. Similarly, trypsin–containing sprays claiming protection against COVID-19 based on a simple trypsin digest [149] are to be avoided, because the protease trypsin has been shown to rather enhance viral invasion and syncytia formation in appropriately designed host models [107, 150]; such sprays sold OTC as medical devices in the European Union are prohibited for sale in Germany because of lack of proof of efficacy.

We propose isotonic rather than hypertonic saline unless for gargling, where hypertonic 2% NaCl is recommended (also by the DGKH). Isotonic saline is devoid of the side effects that we identified throughout this analysis, such as changes in cell morphology and increased nasal epithelial permeability, nasal burning/irritation, and induction of bronchoconstriction or cough. Home-made saline (prepared with simple kitchen salt) is often proposed in the Internet (DGKH: tea spoon flat filled with table/kitchen salt (=2g/100mL corresponding to 1 soup spoon (20 g) in one litre of water). In line with the DGKH, usual applications for airway care are to be carried out 2 to 3 times a day, while ongoing clinical trials in symptomatic COVID-19 patients propose oronasal irrigation up to 12 times per day. Sterile sprays and uni-doses may be safer for nebulizing aerosol. Whether COVID-19 infection only requires (oro)nasal irrigation, or also inhalation of nebulized iso- or hypertonic saline, or whether it can add to other treatment strategies to limit transmission or damage of SARS-CoV-2 to the lungs, deserves further evaluation.

At last, some myths can be debunked. (1) Administration of saline as rinse or aerosol should not be confounded with transmission-prone aerosolizing procedures—a common misconception in respiratory care. Use of oxygen flow or invasive procedures causes the formation of virus-loaded bio-aerosols, by providing air over the infected surfactant-containing surface of the respiratory tract. Saline aerosol in contrast leads to suppression and reduction of exhaled bio-aerosol, as reviewed under Results (point 1). Obviously, saline aerosol and irrigation should be combined with the basic hygiene measures for COVID-19 (see Table 3), with use of disposable or washable tissues to collect superfluous rinse and mucus, with hand washing and adequate room ventilation. (2) Neither is there evidence that nasal rinse would lead to worsening olfaction. Nasal saline irrigation has no effect on normal olfaction [151], and its use in nasal disease has not led to adverse outcome [152]. Its hydrating effect may possibly help to prevent and overcome dry nose during COVID-19 [153].

To summarize, despite the formal effects of saline should be studied in the future by use of (randomized controlled) trials, this review provides sufficient reasoning for the successful application and clinical relevance of saline in the context of mild and early COVID-19. Numerous actions of NaCl relevant to bio-aerosol reduction, primary defence mechanisms such as mucociliary clearance and HOCl production, and to containing SARS-CoV-2 infectivity were identified. These mechanisms are relevant as they are achievable at isotonic (or lower) and hypertonic saline concentrations, used for nasal rinse, respiratory hygiene (aerosol) and oral gargling. They may underlie the traditional use of saline for common cold.

## Supplementary Information

ESM 1 (DOCX 29 kb)

ESM 2 (DOCX 27 kb)

ESM 3 (DOCX 37 kb)

ESM 4 (DOCX 32 kb)

## Abbreviations

ARDS: acute respiratory distress syndrome
ALF: alveolar lining fluid
ASL: airway surface liquid
MCC: mucociliary clearance
ACE: angiotensin converting enzyme
Ang: angiotensin
AT2: alveolar type 2 cells
