# Supplementary material for: Essentials in saline pharmacology for nasal or respiratory hygiene in times of COVID-19
Source: Eur J Clin Pharmacol. 2021 Mar 27;77(9):1275–93. doi: 10.1007/s00228-021-03102-3 (PMC7998085; doi:10.1007/s00228-021-03102-3)
Supplement: Supplementary file 1 — (DOCX 29 kb) [file 228_2021_3102_MOESM1_ESM.docx]

**Essentials in saline pharmacology for nasal or respiratory hygiene in times of COVID-19**

**Supplement 1**. Methods: Approach & Systematic analysis – Key words used on PubMed

A mixed method approach was taken starting with search of local internet (consumer & official sites), followed by structured searches on PubMed with key-words, to end with more broad searches to evaluate or support the relevance to COVID-19.

In an attempt to identify differences in first-line treatment approaches for COVID-19 between Germany [low excess mortality on EUROMOMO (www.euromomo.eu), low hospitalization and case-fatality ratio (www.RKI.de)] and Belgium [high excess mortality and high hospitalization rates and and case-fatality ratio ([www.Sciensano.be](http://www.Sciensano.be))], we searched for online guidance articles on COVID-19 treatment, as accessible to German and Belgian consumers. We combined general key words such as ‘treatment’ and ‘corona/Covid-19’, in Dutch/French and German using the search engines Google.be, Google.de and Yahoo.de. German sources related to ‘AtemwegPflege’ (respiratory care), Aerosol and Kochsalz (kitchen salt) [1-7]. Such information was not found on the Belgian internet. The recommendations of Sciensano (Belgium) [10,11] and the positioning statement of the German lung specialists (pneumologists) [14] were also screened.

We subsequently searched PubMed for literature on saline and sodium chloride (NaCl), resulting in >180,000 articles on saline and >100,000 sources on sodium chloride /NaCl (06/07/2020), obliging us to take mixed method, yet strongly limiting approach. Initially, systematic searches were performed on established properties of saline, thereby combining the key property searched for, with the word saline, both cited in the title. Targeting on ‘saline’ in the title of the searches reduced drastically the number of sources, so some information may have been missed by taking this approach, which was counterchecked through the use of review articles. Only source(s) deemed relevant were selected to qualitatively support the evidence for a given property; those focusing on chronic respiratory disease, such as listed below in the Tables, were not retained, unless if relevant to discriminate effects from a pharmacodynamic, pathophysiological or safety point of view. The retrieved effects of pH, however, were very complex, influencing many processes, such as documented for hypertonic saline. As such, the effects of pH have only been reviewed with regard to (patho) physiological shifts that may be relevant to replication of SARS-CoV-2 in the nose and airways.

While searching for the relevance of these actions of saline to COVID-19 (SARS-CoV-2 virus), additional potential properties of NaCl were identified, leading to further progressive searches on PubMed and Internet in general, using key words relevant to SARS-CoV-2 and linking these with NaCl or saline [no specific keywords or algorithms used].

We addressed in a structured way [see tables below], or qualitative supportive way [only qualitative search to support the claim]:

1. effects on, and risk-benefit evaluation of saline (aerosol) in the formation of bio-aerosol and viral spreading:
   1. for the effects of saline on bio-aerosols (using keywords from vesicle technology such as phospholipid and vesicle) and viral decay, only the most relevant sources relevant to the dynamics on viral stability in droplets or bio-aerosols were retained
   2. for the effects of NaCl on bio-aerosols, ample sources were identified related to pollution, but only 2 sources were selected thought to be relevant to viral stability

[structured search + expanded by supportive searches]

1. effects of saline on the “MCC”, “ciliary beat”, “cilia” including also those related to osmosis, airway surface liquid (ASL), and mucins

[structured search + expanded by supportive searches]

1. the role of saline in mucosal “hydration”

[overlapping with MCC + expanded by supportive search]

1. the role of NaCl in inhibiting viral “replication” including (a) localization and interactions between “sodium chloride” (NaCl or salt) or “saline” and “SARS-CoV-2” with regard to “ACE2” and “ENaC”; (b) inhibition of the host protease “furin” and corona viral protein “3CLpro” (or “Mpro”); (c) pH-effect

[structured + broad supportive search]

1. the role of NaCl in generation of hypochlorous acid and the involvement of myeloperoxidase (MPO) -> [structured search + expanded by supportive searches]
2. supportive evidence for the clinical relevance of these mechanisms, by reviewing relevant clinical results with saline to common colds/upper respiratory symptoms/bronchiolitis or ARDS. To assess the clinical relevance of the mechanisms identified for NaCl, “saline” was searched in combination with “common colds”, “upper respiratory symptoms”, “bronchiolitis”, “ARDS” or COVID-19 (SARS-CoV-2) [structured search].
   1. All studies in COVID-19 are discussed in this study, while only comprehensive sources (meta-analyses and Cochrane) were retained from the vast literature in the other indications.
   2. For the role of saline in prevention of common cold, no review evaluation was available; two large controlled studies (one in children, one in adults) were retrieved.
   3. Evaluations of saline for the management of sinonasal pathology or surgery were not retained.

All review articles identified on saline in COVID-19 were published by companies developing medical devices for nasal irrigation: they contained little, if any, information on pharmacological/pharmacodynamic effects of saline (not cited).

Pathways followed for structured searches and reason for exclusion, unless relevant to the pharmacology/pharmacodynamics in relation to common cold/COVID-19 [July 2020]:

| **Saline (Title) & MCC** | **(saline in title)** | **PubMed : 88 sources** |
| --- | --- | --- |
| ***Excluded*** |  |  |
| Cystic fibrosis | 34 |  |
| Sinonasal disease /surgery | 5 |  |
| Brochiectasis | 4 |  |
| Allergic rhinitis | 6 |  |
| Chronic rhinosinusitis | 5 |  |
| Chronic pulmonary disease (COPD) | 1 |  |
| Animal data | 4 |  |
| Primary ciliary dyskinesia (PCD) | 1 |  |
| Asthma | 3 |  |
| Nasal inflammatory disease/chronic conditions | 2 |  |
| Chronic cough | 1 |  |
| Bronchiolitis | 2 |  |
| **Total** | **68** |  |
| **Adverse events** (retained) |  |  |
| Nasal burning/irritation | 1 |  |
| Cough | 1 |  |
| Epithelial permeability | 2 |  |
| **Other mechanisms** (retained if relevant) |  |  |
| Omosis | 5 |  |
| Absorption in mucus in vitro | 1 |  |
| Rheology of mucus | 1 | (cfr gelling) |
| Reduces secondary infection | 1 |  |
| **Ciliary beat / Mucociliary clearance** (**MCC)** (Retained) |  | **(10 sources)** |
| Isotonic | 5 |  |
| Hypertonic | 4 |  |
| Tissue used = not relevant to CC | 1 |  |
|  | **90** |  |
| (Double use) | -2 |  |
| **Total** | **88** |  |
| **Saline + ciliary beat:** |  | **PubMed : 10 sources** |
| *Excluded:*  Old review 2009 | 1 |  |
| Tissue not representative to common cold (CC)/ upper respiratory tract infection (sphenoidal sinus) | 1 |  |
| Chronic conditions/surgery | 4 |  |
| Comparison seawater (on nasal polyp explants) | 1 |  |
| *Retained:*  Role of heating (retained for discussion) | 1 |  |
| Safety (retained) | 1 |  |
| MCC (retained) | 1 |  |
| **Total** | **10** |  |
| **Saline + Hypochlorous acid + virus:** |  | **PubMed : 17 sources** |
| None relevant to respiratory or CC viruses |  |  |
| **Saline + Hypochlorous acid + virus:** |  | **PubMed : 7 sources** |
| *Excluded:*  not relevant to saline, common cold | 6 |  |
| *Retained:* |  |  |
| Primary nasal epithelial cells | 1 |  |
| **Total** | **7** |  |
| **(NaCl) AND (Myeloperoxidase[Title])**  **(MPO)** | | **PubMed : 25 sources** |
| *Excluded:*  Not directly relevant to saline as intervention for CC/URTI | 24 |  |
| *Retained:* |  |  |
| Needs NaCl for HOCl production | 1 |  |
| **Total** | 25 |  |
| **(Clinical) AND (Saline[Title]) AND (Respiratory)** | | **PubMed : 196 sources** |
| Search reduced to: |  |  |
| **(Clinical) AND (Saline[Title]) AND (Respiratory) AND (Bronchiolitis): PubMed : 29 sources** | | |
| Bronchiolitis |  |  |
| Reviews/meta-analysis | 5 |  |
| ***Excluded:*** |  |  |
| Not supportive, individual studies bronchiolitis | 2 |  |
| Viral wheezing/asthma | 2 |  |
| **Comparison seawater** |  |  |
| Small study | 1 |  |
| **Not relevant or older than Cochrane or meta-analyses** | 19 |  |
| **Total** | **29** |  |
| **(clinical) AND (Saline[Title]) AND (common cold)** | | **PubMed : 11 sources** |
| Retained: | 3 |  |
| *Excluded:* |  |  |
| Older assessments/trials than current reviews | 5 |  |
| Not appropriate | 2 |  |
| Nharinel method | 1 |  |
| **Total** | 11 |  |
| **(Saline) AND (pH)** |  | **PubMed : 4987 sources** |
| Search reduced to: |  |  |
| **(Saline[Title]) AND (pH) AND (nasal)** |  | **PubMed : 77 sources** |
| *Not retained, as not essential to the mechanisms discussed and relevant for common cold (CC) , rather focussing on nasal obstruction and chronic (obstructive)pulmonary diseases:* | | |
| Acute : affects cilia (complex effects relevance to in vivo settingunclear, as electrolyte balance adapts following the pH in the in vitro setting) |  | 1 |
| Residence of saline in nose (patency): 7-35% | | 1 |
| Upregulation chloride channel CLC3 (hypertonic saline) - COPD | | 1 |
| Affects luminal sodium conductance in vitro (hypertonic) - retained | | 1 |
| Refrigeration needed for stock solutions for a test |  | 1 |
| Temperature effect |  | 1 |
| Systemic infusion |  | 3 |
| Nasal reactions to provocative test (no effect of saline) – retained in discussion | | 1 |
| 'Opinion' (unreferenced) |  | 1 |
| Gut irrigation for bowel preparation |  | 1 |
| Case reports (foreign body removal, la) |  | 2 |
| Animal data hypertonic drinking water (ducks) | | 2 |
| Review of effect on interleukins (not related to CC) | | 1 |
| Studies in chronic conditions (relevance unclear to CC), and/or documenting changes following additions of buffers, minerals, drugs or preservatives) |  | 60 |
| **Total** |  | **77** |
| **(Saline) AND (SARS-CoV)** |  | **PubMed 38 sources** |
| Mostly related to preservation/transport of samples | | 26 |
| Ramalingam |  | 1 - Relevant |
| Review - nasal/respiratory care CC symptoms (notCOVID-19) | | 1 |
| Commercial interest, selling medical device | | 2 |
| Hyponatremia during Covid-19 |  | 1 |
| Protocols (saline use during ventilation) |  | 3 |
| Intravenous saline |  | 1 |
| Animal study SARS-COV (not on saline effect) | | 1 |
| Aerosolizing procedure in dentistry during surgery  (not on saline effect) | | 1 |
| Other (stem cell) |  | 1 |
| **SARS-CoV & cilia** |  | **PubMed 12 sources** |
| - not specific enough/not documenting effect on cilia | | 10 |
| COVID-19 & cilia |  | 1 |
| Medical hypothesis on cilia |  | 1 |
| - Broader searches performed ad hoc on SARS-CoV(2) |  |  |
