# Supplementary material for: Essentials in saline pharmacology for nasal or respiratory hygiene in times of COVID-19
Source: Eur J Clin Pharmacol. 2021 Mar 27;77(9):1275–93. doi: 10.1007/s00228-021-03102-3 (PMC7998085; doi:10.1007/s00228-021-03102-3)
Supplement: Supplementary file 2 — (DOCX 27 kb) [file 228_2021_3102_MOESM2_ESM.docx]

**Essentials in saline pharmacology for nasal or respiratory hygiene in times of COVID-19**

**Supplement 2:** Distinguishing bio-aerosols: viral bio-aerosols and bio-aerosol generating procedures.

This section provides some insights on differential aspects when talking about aerosol and consolidates the information from sources that have evaluated the role of ‘nebulising’ (aerosol formed by a nebulizer to administer medicines) in comparison to bio-aerosol producing procedures. Firstly, one should not confound saline aerosol with viral bio-aerosols harvested from cell cultures, as for instance used or referred to in the 2 first examples in the Table, Part A [^[[1]](#endnote-1)^,^[[2]](#endnote-2)^]. The same is valid for bio-aerosol-generating procedures, such as listed by the WHO [^[[3]](#endnote-3)^]. Relevant literature on outcomes of (bio-)aerosol’ generating procedure is listed in the Table, Part B.

Concerns about viral spreading by (nebulizing) aerosol has been mainly raised following the report of a hospital case of SARS-CoV-1 in Hong Kong, associated with contamination in a ward following 7 days of nebulizing salbutamol [^[[4]](#endnote-4)^]. Yet, as reviewed in the Table, several subsequent independent evaluations have not found a significant effect of nebulizing treatment on transmission [^[[5]](#endnote-5)^-,^[[6]](#endnote-6)^,^[[7]](#endnote-7)^,^[[8]](#endnote-8)^,^[[9]](#endnote-9)^]. Recommendations with regard to the use of nebulization/aerosol treatment are discrepant across countries. Although the KCE (Belgian Health Care Knowledge Centre) identified no enhanced transmission risks of saline aerosol nebulizer treatment [7], saline aerosol use is discouraged in Belgium, unless in an isolated home situation or in the open air; users should then ventilate the room for a minimum of 30 minutes after the atomization [^[[10]](#endnote-10)^].This contrasts to the German situation, as an early German position paper by pneumologists on COVID-19 [^[[11]](#endnote-11)^] refers to two retrospective analyses regarding the procedure-related risk of nebulizer applications that were carried out during the SARS epidemic in Canada in 2003: these could not establish an increased risk of infection for the medical personnel [8,9]. Also the British Thoracic Society states in his ‘advice about the safety of nebuliser’ use that “Advice from PHE and HPS is that nebulisation is not a VIRAL droplet generating procedure. The droplets are from the machine (liquid bronchodilator drug particles), not the patient. Nebulisation is not therefore considered a 'viral' aerosol generating procedure.” [^[[12]](#endnote-12)^].

Conclusion: Bio-aerosol-generating procedures, such as intubation, extubating and related procedures, prone positioning, disconnecting patient from ventilator, tracheotomy/ tracheostomy manipulation, manual ventilation, open suctioning, bronchoscopy or non-invasive ventilation, implicated in high risks of SARS-CoV-2 transmission, should not be confounded with aerosols generated by a nebulizing device. The latter procedure has not been associated with enhanced risk of SARS-CoV-2 infection.

**Table 1. Effects of wetting, nebulizing and rinsing on saline in viral transmission**

| Report/Study of | Procedure | Results & (Proposed) mechanism | Ref. |
| --- | --- | --- | --- |
| **Virus containing bio-aerosol or aliquots *in vitro* – without saline** | | | |
| Bio-aerosol in culture medium | Nebulizing viable viral culture on various surfaces | Survival of SARS-CoV-2 virus in bio-aerosol, yet originating from a nebulised virus-growing culture medium as carrier | van Doremalen et al. 2020 [1] |
| Bio-aerosol review | Hypothesis built on studies with various types of aerosols | - Bio-aerosols are generated in the deep lung through reopening of collapsed small airways during inspiration - Deposition of inhaled 0.1–0.5 µm particles is only 30% -70% of inhaled particles are exhaled again | Scheuch et al. 2020 [2] |
| **Bio-aerosol-generating procedures in hospital care** | | | |
| Bio-aerosol-generating procedures | Intubation, extubating and related procedures, prone positioning, disconnecting patient from ventilator, tracheotomy/ tracheostomy manipulation, manual ventilation, open suctioning, bronchoscopy or non-invasive ventilation | Viral spread by invasive procedures causing basal/airway damage and spread of surfactant-containing ALF is being referred to | WHO [3] |
| Nebulised salbutamol | Hong Kong hospital case report with SARS-CoV - Aerosol use with salbutamol – 0.5 mg through jet nebuliser, delivered by oxygen at a flow rate of 6 L/min, 4/day,7 days. | Association with contamination in a ward following 7 days of nebulizing salbutamol | Lee et al. 2003 [4] |
| Bio-aerosol-generating procedures | Systematic review of transmission of acute respiratory infections to healthcare workers | Nebuliser treatment found to be not significant | Tran et al. 2012 [5] |
| Bio-aerosol-generating procedures | Evaluation of infective risk to healthcare workers for SARS-CoV-2 | Little evidence detailing the transmission of SARS-CoV-2 associated with any specific procedures. | Harding et al. 2020 [6] |
| Bio-aerosol-generating procedures | Assessment of various aerosol-generating procedures | No enhanced transmission risks of saline nebuliser treatment identified | Jespers at al. KCE, Belgium, 2020 [7] |
| Bio-aerosol-generating procedures | Evaluation of nebuliser applications during SARS epidemic 2003 in Canada | No increased risk of infection for medical staff with use of nebulisers | Raboud et al. 2010 [8] |
| Bio-aerosol-generating procedures | Evaluation of nebuliser applications during SARS epidemic 2003 in Canada | No increased risk of infection for medical staff with nebulisers | Loeb et al. 2004 [9] |

**References**

1. van Doremalen N, Morris DH, Holbrook MG, Holbrook MG, Gamble A, Williamson BN (2020) Aerosol and surface stability of SARS-CoV-2 as compared with SARS-CoV-1. N Engl J Med 382:1564-7. https://doi.org/10.1056/NEJMc2004973 [↑](#endnote-ref-1)
2. Scheuch G (2020) Breathing is enough: for the spread of influenza virus and SARS-CoV-2 by breathing only. J Aerosol Med Pulm Drug Delivery 33:230-4. doi.org/10.1089/jamp.2020.1616 [↑](#endnote-ref-2)
3. World Health Organization (2020) Modes of transmission of virus causing COVID-19: implications for IPC precaution recommendations. Scientific brief, 29 March 2020. <https://www.who.int/publications-detail/modes-of-transmission-of-virus-causing-covid-19-implications-for-ipc-precaution-recommendations>. Accessed June 19, 2020 [↑](#endnote-ref-3)
4. Lee N, Hui D, Wu A, Chan P et al (2003) A major outbreak of severe acute respiratory syndrome in Hong Kong. N Engl J Med 348:1986e94. <https://doi.org/10.1056/NEJMoa030685> [↑](#endnote-ref-4)
5. Tran K, Cimon K, Severn M, Pessoa-Silva CL, Conly J (2012) Aerosol generating procedures and risk of transmission of acute respiratory infections to healthcare workers: a systematic review. PLOS ONE 7:e35797. <https://doi.org/10.1371/journal.pone.0035797> [↑](#endnote-ref-5)
6. Harding H, Broom A, Broom J (2020) Aerosol-generating procedures and infective risk to healthcare workers from SARS-CoV-2: the limits of the evidence. J Hospital Infection 105:717-725. https://doi.org/<https://doi.org/10.1016/j.jhin.2020.05.037> [↑](#endnote-ref-6)
7. Jespers V, Roberfroid D (2020) COVID-19 – KCE Contributions. Aerosol-generating procedures. <https://kce.fgov.be/sites/default/files/atoms/files/2020-51_COVID_Aerosol%20KCE_FINAL_19052020_3.pdf> [↑](#endnote-ref-7)
8. Raboud J, Shigayeva A, McGeer A et al (2010) Risk factors for SARS transmission from patients requiring intubation: a multicentre investigation in Toronto, Canada. PLOS ONE 5(5):e10717. <https://doi.org/10.1371/journal.pone.0010717> [↑](#endnote-ref-8)
9. Loeb M, McGeer A, Henry B et al (2004) SARS among critical care nurses, Toronto. Emerg Infect Dis 10(2):251–5. <https://doi.org/10.3201/eid1002.030838> [↑](#endnote-ref-9)
10. APB (2020) Aerosoltoestellen [Aerosol devices]. Information Update 20 March 2020. <https://www.apb.be/APB%20Documents/NL/All%20partners/CORONAVIRUS_AEROSOL_VERHUUR_20_03_20.pdf>. Accessed 19 June 2020. [↑](#endnote-ref-10)
11. Pfeifer M, Ewig S, Voshaar T et al (2020) Position paper for the state-of-the-art application of respiratory support in patients with COVID-19. Respiration 99:521–41. <https://doi.org/10.1159/000509104> . [↑](#endnote-ref-11)
12. British Thoracic Society (2020) Advice about the safety of nebuliser use (Last update 23/3/20). <https://www.brit-thoracic.org.uk/covid-19/covid-19-information-for-the-respiratory-community/#advice-about-the-safety-of-nebuliser-use> Accessed 17 January 2020 [↑](#endnote-ref-12)
