# Supplementary material for: Essentials in saline pharmacology for nasal or respiratory hygiene in times of COVID-19
Source: Eur J Clin Pharmacol. 2021 Mar 27;77(9):1275–93. doi: 10.1007/s00228-021-03102-3 (PMC7998085; doi:10.1007/s00228-021-03102-3)
Supplement: Supplementary file 3 — (DOCX 37 kb) [file 228_2021_3102_MOESM3_ESM.docx]

**Essentials in saline pharmacology for nasal or respiratory hygiene in times of COVID-19**

**Supplement 3:** Sodium chloride (NaCl) and myeloperoxidase activity (MPO)

This section lists a selection of sources to illustrate the complex interactions between NaCl, chloride (Cl^-^) in particular, and the MPO activity. Inhibition of viral replication in presence of chloride and halide salts was first reported in the 1960s: hypochlorous acid (HOCl) has the well-known effect of bleach, being effective against all virus types for house-hold purposes. Yet, HOCl is also cytotoxic and may injure airway epithelial cells *in vitro* [^[[1]](#endnote-1)^]. Apart from HOCl production, there are many more complex interactions between NaCl and MPO, involving also other substrates.

The main mechanisms as we identified relevant for MPO in viral infection and involving NaCl are listed in Table A. These involve HOCl formation, hypothiacianate production, formation of reactive oxygen species (ROS) and extracellular neutrophil traps (NETs) formation.

These interactions all can help to kill pathogens, suggesting that the mechanisms by MPO are part of the innate antiviral immune mechanism to clear viral and bacterial infections [^[[2]](#endnote-2)^,^[[3]](#endnote-3)^]. Several, if not all, of these mechanisms may also be relevant to SARS-CoV-2 viral infection.

While generation of oxidants by MPO is beneficial in terms of the immune response to invading pathogens, inappropriate formation of oxidants (at the wrong place, the wrong time or at excessive levels) is likely to result in host tissue damage [^[[4]](#endnote-4)^].

Relevance to lung disease & SARS-CoC-2:

Human MPO activity is involved in phagocytosis by neutrophils and macrophages and in oxidative processes, as well as their feedback mechanisms, in the bronchi and lungs [^[[5]](#endnote-5)^-,^[[6]](#endnote-6)^,^[[7]](#endnote-7)^,^[[8]](#endnote-8)^]. Four-fold enhanced MPO activity in airway fluid is associated with infections in the airways of children with cystic fibrosis compared to those without respiratory infection [^[[9]](#endnote-9)^].

The role of MPO in SARS-CoV-2 is unclear. A number of sources illustrate this in Table B. To summarise: on one hand, MPO increase would be a marker for early disease. On the other hand, it has been found to be increased in mechanically ventilated COVID-19 patients. MPO would contribute to many oxidative processes involved in the deterioration and fatality in COVID-19. A complex hypothesis implicates MPO in the damage associated with overwhelming inflammation during COVID-19 (see Table B).

Conclusion:

The complex regulation of the MPO pathways demands a separate in-depth study as to evaluate the impact of saline, also in relation of the contribution of MPO with regard to SARS-CoV-2 infection.

[To note: direct exposure of cilia to HOCl has been found to cause ciliostasis, which may possibly contribute to discrepant effects obtained with hypertonic saline on the MCC between different studies [^[[10]](#endnote-10)^]. So, tight regulation of this metabolic route is needed.]

Suppl. 3 - Table A:

| **Source** | **Selection of sources to interpret interactions between NaCl and MPA** |
| --- | --- |
|  | 1. **Production od HOCl:** NaCl may produce HOCl via MPO |
| Speir et al. 1961 [2] | Viral inactivation was observed at NaCl concentrations between 15-300 mM (corresponding to 0.09%-1.7% of saline) and appeared to be related to the presence of Cl^-^ or similar anions, rather than Na^+^. |
| Ramalingam et al 2018 [^[[11]](#endnote-11)^] | Viral inactivation of DNA, RNA, enveloped and non-enveloped viruses, including the human coronavirus 229E (HCoV-229E):   - Viral inhibition was dose-dependent and measurable from 10 mM NaCL (0.058%) onwards, as well as dependent on the virus tested. - The *in vitro* effect was not due to a direct effect of NaCl on the host cells, but happened during viral replication |
| Suzuki et al. [^[[12]](#endnote-12)^] | Neutrophil MPO activity increases with increasing NaCl concentrations from 0.025 to 0.14 M (0.14%-0.82%).  PH and Cl^‑^ concentration determines MPO activity: Vmax values of MPO-I, -II, and -III are also higher at pH 4.9 and 5.4 and further increased with increasing NaCl concentration. |
| Wang et al. 2015 [3] | HOCl generation in the phagosomes requires a continuous supply of chloride: the local chloride disposition drives chloride redistribution into the neutrophil phagosomes by various mechanisms and sustains HOCl production  [NaCl may make chlorination dominating over peroxidation, producing HOCl with higher antimicrobial action than H_2_O_2._] |
| Zhang et al. 2013 [^[[13]](#endnote-13)^]. | MPO activity is present in lung tissue.  Adding NaCl to cell culture medium *in vitro* may shift the MPO activity to production of different metabolites. |
|  | 1. **Thiocyanate – hypothiocyanite production**   Alternatively, Cl^-^ competes with thiocyanate as natural substrate for MPO activity. Saline may also shift the substrate thiocyanate towards alternative signalling pathways, (or enhance its local availability), thereby exerting host defence and antioxidant properties, in addition to the effects of HOCl |
| Dalen et al. 1997 [^[[14]](#endnote-14)^] | Chloride is considered to be the physiological substrate of neutrophil MPO: while MPO uses H_2_O_2_ to oxidize chloride and thiocyanate to their respective hypohalous acids, substrate preference is influenced by chloride at physiological concentrations of these substrates.   - Thiocyanate was found to be by far the most favoured substrate for MPO, producing hypothiocyanite at concentrations of thiocyanate as low as 25 mM, in the presence of 100 mM chloride. - MPO was more active in the presence of thiocyanate and chloride than with either of these substrates alone. |
| Gould et al. 2010  [^[[15]](#endnote-15)^] | Hypertonic saline increases glutathione and thiocyanate in the lung epithelial lining fluid (ELF).   - This is in vitro associated with protective effects in the ELF against HOCl toxicity - Glutathione and thiocyanate levels found in the ELF are sufficient to protect against HOCl mediated toxicity in vitro. |
| Chandler et al. 2012 [^[[16]](#endnote-16)^] | To note: isothiocyanate is used in TRIzol to inactivate SARS-CoV-2 for extraction and qPCR analysis. |
| Darnell et al. 2010 [^[[17]](#endnote-17)^] | 1. **Reactive oxygen species (ROS) & extracellular neutrophil traps (NETs**)   The NaCl concentration may tightly regulate MPO-activity and its ensuing processes: these involve the formation of ROS. ROS can kill pathogens directly by causing oxidative damage or indirectly, in neutrophils, by stimulating pathogen elimination via extracellular neutrophil trap (NET) formation [23]. ROS can however have a detrimental role, promoting venous thrombus formation through the modulation of the enzymatic cascade of fibrinolysis systems of coagulation and the complement system: NETosis can have a detrimental effect in cardiovascular and pulmonary diseases [23]. |
| Nadesalingam et al. 2018 [^[[18]](#endnote-18)^] | Hypertonic saline has been shown to suppress the NADPH oxidase-dependent neutrophil extracellular trap (NET) formation while it promotes apoptosis:   - phorbol myristate acetate (PMA)-mediated NETosis was observed after 120 min upon use of isotonic and lower NaCl concentrations (100-150 mM). - The NETosis was increasingly supressed by increasing NaCl concentrations >150 mM – 509 mM. - Hypertonic saline also suppressed the production of LPS- and PMA-induced ROS. - The effect would be effectuated by neutrophil dehydration, while the intracellular pH was not significantly affected. - The suppression of NETotic cell death was coupled with a promotion of the apoptotic pathway of cell death. - Hence, under a hypertonic saline condition, neutrophils undergo apoptosis instead of only NETosis. |
| Papayannopoulos et al. 2010 [^[[19]](#endnote-19)^] | This study shows very complex neutrophil interactions between MPO with NET, DNA and NaCl. |

Suppl. 3 – Table B:

| **Source** | **Interaction between SARS-CoV-2 and MPA** |
| --- | --- |
| Guéant et al. 2020 [^[[20]](#endnote-20)^] | Blood level of MPO‐DNA complexes are enhanced in COVID-19. Their level could be a useful biomarker of early SARS‐CoV‐2 infection, as also present in asymptomatic patients. |
| Zhuo [^[[21]](#endnote-21)^] | MPO is increased in sera of COVID-19 patients, particularly if mechanically ventilated. Neutrophil extracellular traps (NETs) are extracellular webs of chromatin, microbicidal proteins, and oxidant enzymes that are released by neutrophils to contain infections. NETs may not be properly regulated in COVID-19 disease. |
| Akgun et al. 2020  [^[[22]](#endnote-22)^] | Significant MPO levels are identified in naso-oropharyngeal samples of SARS-CoV-2 patients: these are associated with changes in also other proteins and suggest specific activation of the innate immune system via neutrophil degranulation and the NETs. |
| Arcanjo et al. 2020 [^[[23]](#endnote-23)^] | SARS-CoV-2 is able to activate NETosis in human neutrophils and increases levels of intracellular ROS in neutrophils. |
| Goud et al. 2020 [^[[24]](#endnote-24)^] | COVID-19 related mortality is characterised by multi-system involvement, generalized tissue damage, vasoconstriction, severe hypoxia and rapid clinical deterioration in critically ill COVID-19 patients. Proposed hypothesis:   - Reactive oxygen species (ROS) may be activated. ROS may result in consumption of nitric oxide, a critical vasodilation regulator. - Activated neutrophils are known to release MPO during inflammation/infection as part of the natural immune response, which contributes to production of hypochlorous acid (HOCl).. - It is proposed that, if overwhelming inflammation, HOCl competes with O_2_ at heme binding sites, decreasing O_2_ saturation, and contributes to oxidative reactions, such as the hemoglobin-heme iron oxidation, heme destruction, and subsequent release of free iron [Free iron mediates toxic tissue injury through additional ROS generation and NO consumption] |

References Supplement 3

1. Regelmann WE, Schneider LA, Fahrenkrug SC et al. (1997) Proteinase-free myeloperoxidase increases airway epithelial permeability in a whole trachea model. Pediatr Pulmonol 24(1):29-34. <https://doi.org/10.1002/(sici)1099-0496(199707)> [↑](#endnote-ref-1)
2. Speir RW (1961) Effect of several inorganic salts on the infectivity of Mengo virus. Proc Soc Exp Biol Med 106:402–404. <https://doi.org/10.3181/00379727-106-26352> [↑](#endnote-ref-2)
3. Wang G, Nauseef WM (2015) Salt, chloride, bleach, and innate host defense. J Leukocyte Biol 98(2): 163–72. <https://doi.org/10.1189/jlb.4RU0315-109R> [↑](#endnote-ref-3)
4. Davies MJ (2011) Myeloperoxidase-derived oxidation: mechanisms of biological damage and its prevention. J Clin Biochem Nutr 48(1):8-19. <https://doi:10.3164/jcbn.11-006FR> [↑](#endnote-ref-4)
5. Klebanoff SJ, Kettle AJ, Rosen H et al (2013) Myeloperoxidase: a front-line defender against phagocytosed microorganisms. J Leukoc Biol 93(2):185-98. <https://doi.org/10.1189/jlb.0712349> [↑](#endnote-ref-5)
6. Haegens A, Vernooy JHJ, Heeringa P et al (2008) Myeloperoxidase modulates lung epithelial responses to pro-inflammatory agents. Eur Respiratory J 31:252-260. <https://doi.org/10.1183/09031936.00029307> [↑](#endnote-ref-6)
7. Casciaro M, Di Salvo E, Pace E et al (2017) Chlorinative stress in age-related diseases: a literature review. Immun Ageing 14:21. <https://doi.org/10.1186/s12979-017-0104-5> [↑](#endnote-ref-7)
8. Khan AA, Alsahli MA, Rahmani AH (2018) Myeloperoxidase as an active disease biomarker: recent biochemical and pathological perspectives. Med Sci (Basel) 6(2):33. <https://doi.org/10.3390/medsci6020033> [↑](#endnote-ref-8)
9. Kettle AJ, Chan T, Osberg I et al. (2004) Myeloperoxidase and protein oxidation in the airways of young children with cystic fibrosis. Am J Respir Crit Care Med 170(12):1317-23. <https://doi.org/10.1164/rccm.200311-1516OC> [↑](#endnote-ref-9)
10. Kasahara K, Kawakami Y, Kiyono T et al. (2014) Ubiquitin-proteasome system controls ciliogenesis at the initial step of axoneme extension. Nat Commun 5:5081. <https://doi.org/10.1038/ncomms6081> [↑](#endnote-ref-10)
11. Ramalingam S, Cai B, Wong J et al. (2018) Antiviral innate immune response in non-myeloid cells is augmented by chloride ions via an increase in intracellular hypochlorous acid levels. Sci Rep 8:13630. <https://doi.org/10.1038/s41598-018-31936-y> [↑](#endnote-ref-11)
12. Suzuki K, Yamada M, Akashi K, Fujikura T (1986) Similarity of kinetics of three types of myeloperoxidase from human leukocytes and four types from HL-60. Arch Biochem Biophysics 245(1):167-73. <https://doi.org/10.1016/0003-9861(86)90201-8> [↑](#endnote-ref-12)
13. Zhang N, Francis KP, Prakash A, Ansaldi D (2013) Enhanced detection of myeloperoxidase activity in deep tissues through luminescent excitation of near-infrared nanoparticles. Nat Med 19(4):500-5. <https://doi.org/10.1038/nm.3110> [↑](#endnote-ref-13)
14. van Dalen CJ, Whitehouse MW, Winterbourn CC, Kettle AJ (1997) Thiocyanate and chloride as competing substrates for myeloperoxidase. Biochem J. 327:487-92. <https://doi:10.1042/bj3270487> [↑](#endnote-ref-14)
15. Gould NS, Gauthier S, Kariya CT et al (2010) Hypertonic saline increases lung epithelial lining fluid glutathione and thiocyanate: two protective CFTR-dependent thiols against oxidative injury. Respir Res 11(1):119. <https://doi:10.1186/1465-9921-11-119> [↑](#endnote-ref-15)
16. Chandler JD, Day BJ (2012) Thiocyanate: a potentially useful therapeutic agent with host defense and antioxidant properties. Biochem Pharmacol 84(11):1381-7. <https://doi.org/10.1016/j.bcp.2012.07.029> [↑](#endnote-ref-16)
17. Darnell ME, Subbarao K, Feinstone SM, Taylor DR (2004) Inactivation of the coronavirus that induces severe acute respiratory syndrome, SARS-CoV. J Virol Methods 121(1):85-91. <https://doi.org/10.1016/j.jviromet.2004.06.006> [↑](#endnote-ref-17)
18. Nadesalingam A, Chen JHK, Farahvash A, Khan MA (2018) Hypertonic Saline Suppresses NADPH Oxidase-Dependent Neutrophil Extracellular Trap Formation and Promotes Apoptosis. Front Immunol 9:359. <https://doi:10.3389/fimmu.2018.00359> [↑](#endnote-ref-18)
19. Papayannopoulos V, Metzler KD, Hakkim A, Zychlinsky A (2010) Neutrophil elastase and myeloperoxidase regulate the formation of neutrophil extracellular traps. J Cell Biol 191(3):677-91. <https://doi:10.1083/jcb.201006052> [↑](#endnote-ref-19)
20. Guéant JL, Fromonot J, Guéant-Rodriguez RM et al (2020) Blood myeloperoxidase-DNA, a biomarker of early response to SARS-CoV-2 infection? Allergy :10.1111/all.14533. https://doi:10.1111/all.14533. [↑](#endnote-ref-20)
21. Zuo Y, Zuo M, Yalavarthi S et al. (2020) Neutrophil extracellular traps in COVID-19. JCI Insight 5(11):e138999. <https://doi.org/10.1172/jci.insight.138999> [↑](#endnote-ref-21)
22. Akgun E, Tuzuner MB, Sahin B et al. (2020) Proteins associated with neutrophil degranulation are upregulated in nasopharyngeal swabs from SARS-CoV-2 patients. PLoSONE 15(10):e0240012. <https://doi.org/10.1371/journal.pone.0240012> [↑](#endnote-ref-22)
23. Arcanjo A, Logullo J , Menezes CCB et al (2020). The emerging role of neutrophil extracellular traps in severe acute respiratory syndrome coronavirus 2 (COVID-19). Sci Rep 10:19630 <https://doi.org/10.1038/s41598-020-76781-0> [↑](#endnote-ref-23)
24. Goud PT, Bai D, Abu-Soud HM (2021) A Multiple-Hit Hypothesis Involving Reactive Oxygen Species and Myeloperoxidase Explains Clinical Deterioration and Fatality in COVID-19. Int J Biol Sci 17(1):62-72. doi:10.7150/ijbs.51811. <https://www.ijbs.com/v17p0062.htm> [↑](#endnote-ref-24)
