# Supplementary material for: Essentials in saline pharmacology for nasal or respiratory hygiene in times of COVID-19
Source: Eur J Clin Pharmacol. 2021 Mar 27;77(9):1275–93. doi: 10.1007/s00228-021-03102-3 (PMC7998085; doi:10.1007/s00228-021-03102-3)
Supplement: Supplementary file 4 — (DOCX 32 kb) [file 228_2021_3102_MOESM4_ESM.docx]

**Essentials in saline pharmacology for nasal or respiratory hygiene in times of COVID-19**

**Supplement 4:** Translation of the mechanisms into clinical benefits

| 1. **Saline aerosol in bronchiolitis** |
| --- |

Acute bronchiolitis in children – Isotonic saline

Based on a meta-analysis of studies in non-COVID-19-associated ARDS, isotonic saline aerosol (0.9%, also called physiologic serum) has been proposed as an active treatment for acute viral bronchiolitis, rather than an inert placebo [^[[1]](#endnote-1)^,^[[2]](#endnote-2)^]: patients with viral bronchiolitis treated with nebulized normal saline showed significant improvement in the respiratory rate, clinical scores after therapy and reduced hospital length of stay by 24h. Tolerance in infants was excellent. When comparing “saline placebo” with other (non-drug containing) placebos, patients treated with nebulized isotonic saline showed greater improvements in posttreatment scores [1,2].

Acute bronchiolitis in children – Hypertonic saline

In a Cochrane analysis of studies with nebulised hypertonic saline and a meta-analysis [^[[3]](#endnote-3)^,^[[4]](#endnote-4)^], it was concluded that hypertonic saline use may modestly reduce length of stay among infants hospitalised with acute bronchiolitis and improve the clinical severity score. Furthermore, treatment with nebulised hypertonic saline was found to reduce the risk of hospitalisation among outpatients and emergency department patients. Yet, in a large direct comparative study in infants, nebulized hypertonic saline did less well than isotonic saline, while worsening of cough occurred more frequently among children in the hypertonic saline group [^[[5]](#endnote-5)^]. Also other studies failed to confirm a clinical benefit of hypertonic saline on the length of stay or readiness for discharge when compared to normal saline/care [^[[6]](#endnote-6)^,^[[7]](#endnote-7)^].

| 1. **Nasal irrigation/rinse with saline for common cold/** **upper respiratory tract infection (URTI)** |
| --- |

Saline spray/irrigation, ‘normal saline’ – Children & Adults

A 2014 Cochrane analysis (544 children and 205 adult) found limited data from five randomised controlled trials (RCTs) suggesting that saline nasal irrigation may have some benefit in patients with acute upper respiratory tract infections, but the included trials were generally small and were found to carry a high risk of bias [^[[8]](#endnote-8)^]. The evaluation screened all studies using atomised sprays or irrigation with larger volumes of saline solutions, and all types of commercially available saline preparations and concentrations, including isotonic and hypertonic solutions; yet, the number of well conducted studies was low. While some participants experienced minor discomfort, no serious side effects were identified.

Saline strength not specified (‘normal saline’) - Children

A recent meta-analysis of saline nasal irrigation for acute upper respiratory tract infections in infants and children showed that saline significantly improved rhinological symptoms, but not respiratory symptoms [^[[9]](#endnote-9)^]. Its use, however, appeared to reduce the use of other treatments, whether local or systemic, and particularly antibiotics. Long-term use led to a decrease in the incidence of acute rhinosinusitis and its complications. [Saline strength was not specified].

Isotonic saline basal spray – Children & Adults

Studies of isotonic saline in common colds have shown a reduction in the number of illness days and infectious episodes in adults and children, including substantially reduced absence from school (17% vs 35%) and secondary medical complications (8% vs 32%) in children [^[[10]](#endnote-10)^,^[[11]](#endnote-11)^].

Regarding nasal irrigation with saline, the WHO has only recently acknowledged that use of saline may promote recovery from common cold; they however state that to date, there is no evidence that it can protect people from infectious respiratory diseases or COVID‑19 [^[[12]](#endnote-12)^,^[[13]](#endnote-13)^].

| 1. **URTI/ARDS of SARS-CoV-2 and other human coronaviruses** |
| --- |

Nasal rinse with hypertonic saline – Adults – URTI coronavirus/COVID-19

Regarding upper respiratory infections of SARS-CoV-2 and other human coronaviruses, clinical evidence start to compile, yet remains scanty. A recent publication called for the use of hypertonic saline nasal irrigation and gargling as a treatment option, based on prior data generated from a randomised pilot study: this study was performed in 68 patients with an upper respiratory tract infection, of whom 56% were infected by rhinovirus and 31% by “common” (non-COVID-19) coronaviruses [^[[14]](#endnote-14)^]. Patients were enrolled within 48 hours of onset of symptoms; the parameters were rated for a maximum of 14 days or until patients felt well for two consecutive days. In this study, the rinse procedure (gargling =/- nasal rinse) was proposed to act through formation of hypochlorous acid, and allowed to be used maximum 12 times/day; the NaCl concentration was 3%. The hypertonic saline rinses reduced the duration of upper respiratory tract infection by an average of 1.9 days (P = 0.01), the transmission within household contacts by 35% (P = 0.006) and 30% more individuals had reduction in viral shedding by ≥0.5 log10 per day in the intervention arm, as compared to controls. The relevance of these data in the context of COVID-19 needs confirmation.

A study in non-morbid COVID-19 outpatients by the same investigators group of the first study is ongoing including the use of hypertonic saline nasal irrigation up to 12 times daily in addition to standard hygiene and social distancing recommendations [^[[15]](#endnote-15)^,^[[16]](#endnote-16)^].

Nasal rinse with hypertonic saline – Adults – URTI by COVID-19

A recent interim analysis of a small open-labelled study in 45 non-hospitalised COVID‑19 patients in the United States suggests substantial symptom resolution, with nasal saline or saline plus detergent irrigation: nasal congestion and headache resolved 7-9 days (median) earlier in irrigation groups: the viral load data and results in the planned 90 patients are awaited [^[[17]](#endnote-17)^,^[[18]](#endnote-18)^].

Nasal rinse with saline [strength not defined] – Adults – COVID-19 with anosmia

Another prospective study in 45 COVID-19 patients, receiving usual medical care plus intravenous or nebulised electrolysed saline, compared the outcomes with 39 patients in the control group (usual medical care alone) [^[[19]](#endnote-19)^] The process of electrolysis would form HOCl. The intervention led to a decrease of hospitalisation by 92% (p=0.02), a faster acceptable symptom status after 4.6 days on average (instead of 11.0 days in the control group, p=0.015) and reduced mortality (0 versus 12.8% (p=0.019), while all of the 10 qPCR followed patients were COVID-19 negative by day 9. Nebulization was mentioned to be less efficacious than its combination with its intravenous administration. The outcome of simple saline nebulisation was not reported. In a study in COVID-19 patients with anosmia without nasal obstruction, 57% received saline irrigation as initial treatment: smell recovery was noted, albeit slow or partial in part of them, but none of the patients were hospitalised or developed ARDS [^[[20]](#endnote-20)^]. Recovery was not reported by treatment group..

Isotonic saline aerosol – ICU adults with COVID-19 ARDS

A German study of saline nebulisation (to reduce bio-aerosol) prior to (bio-aerosol inducing) non-invasive ventilation procedures, in ICU patients for COVID-19 ADRS, is reported in the internet. Recovery was observed in 57 of the 60 patients without need for invasive ventilation. The three patients who required intubation all had significant comorbidity: one patient died. The study used an advanced hygiene concept: none of health care personnel in the hospital (~1600 persons) became infected [^[[21]](#endnote-21)^].

**References – Supplement 4**

1. House SA, Gadomski AM, Ralston SL (2020) Evaluating the placebo status of nebulized normal saline in patients with acute viral bronchiolitis. A systematic review and meta-analysis. JAMA Pediatr 174(3):250-259. <https://doi.org/10.1001/jamapediatrics.2019.5195> [↑](#endnote-ref-1)
2. Sauvaget E, David M, Bresson V et al (2012) Sérum salé hypertonique nébulisé et bronchiolite aiguë du nourrisson : données actuelles [Nebulized hypertonic saline and acute viral bronchiolitis in infants: current aspects]. Arch Pediatr 19(6):635-41. <https://doi.org/10.1016/j.arcped.2012.03.018> [↑](#endnote-ref-2)
3. Zhang L, Mendoza-Sassi RA et al (2017) Nebulised hypertonic saline solution for acute bronchiolitis in infants. Cochrane Database Syst Rev12(12):CD006458. <https://doi.org/10.1002/14651858.CD006458.pub4> [↑](#endnote-ref-3)
4. Hsieh CW, Chen C, Su HC, Chen KH (2020) Exploring the efficacy of using hypertonic saline for nebulizing treatment in children with bronchiolitis: a meta-analysis of randomized controlled trials. BMC Pediatr 20(1):434. <https://doi.org/10.1186/s12887-020-02314-3> [↑](#endnote-ref-4)
5. Angoulvant F, Bellêttre X, Milcent K et al (2017) Effect of nebulized hypertonic saline treatment in emergency departments on the hospitalization rate for acute bronchiolitis: a randomized clinical trial. JAMA Pediatr 171(8):e171333. <https://doi.org/10.1001/jamapediatrics.2017.1333> [↑](#endnote-ref-5)
6. Everard ML, Hind D, Ugonna K et al (2015) Saline in acute bronchiolitis RCT and economic evaluation: hypertonic saline in acute bronchiolitis - randomised controlled trial and systematic review. Health Technol Assess 19(66):1-130. <https://doi.org/10.3310/hta19660> [↑](#endnote-ref-6)
7. Morikawa Y, Miura M, Furuhata MY et al (2018) Tokyo Pediatric Clinical Research Network. Nebulized hypertonic saline in infants hospitalized with moderately severe bronchiolitis due to RSV infection: A multicenter randomized controlled trial. Pediatr Pulmonol 53(3):358-365. <https://doi.org/10.1002/ppul.23945> [↑](#endnote-ref-7)
8. King D, Mitchell B, Williams CP, Spurling GKP (2015) Saline nasal irrigation for acute upper respiratory tract infections. Cochrane Database of Systematic Reviews Issue 4. Art. No.: CD006821. <https://doi.org/10.1002/14651858.CD006821.pub3> [↑](#endnote-ref-8)
9. Cabaillot A, Vorilhon P, Roca M et al (2020) Saline nasal irrigation for acute upper respiratory tract infections in infants and children: A systematic review and meta-analysis. Paediatr Respir Rev:S1526-0542(20)30016-6. <https://doi.org/10.1016/j.prrv.2019.11.003> [↑](#endnote-ref-9)
10. Slapak I, Skoupa J, Strnad P, Hornik P (2008) Efficacy of isotonic nasal wash (seawater) in the treatment and prevention of rhinitis in children. Arch Otolaryngol Head Neck Surg 134:67–74. <https://doi.org/10.1001/archoto.2007.19> [↑](#endnote-ref-10)
11. Tano L, Tano KA (2004) Daily nasal spray with saline prevents symptoms of rhinitis. Acta Otolaryngol 124 (9) 1059- 62. <https://doi.org/10.1080/00016480410017657> [↑](#endnote-ref-11)
12. WHO (2020) Saline. <https://www.who.int/emergencies/diseases/novel-coronavirus-2019/advice-for-public/myth-busters#saline> [↑](#endnote-ref-12)
13. WHO (2020) Can rinsing your nose regularly with saline solution prevent Covid-19? <https://www.who.int/docs/default-source/searo/thailand/12myths-final099bfbf976c54d5fa3407a65b6d9fa9d.pdf> [↑](#endnote-ref-13)
14. Ramalingam S, Graham C, Dove J et al (2019). A pilot, open labelled, randomised controlled trial of hypertonic saline nasal irrigation and gargling for the common cold. Sci Rep 9:1015. <https://doi.org/10.1038/s41598-018-37703> [↑](#endnote-ref-14)
15. The University of Edinburgh (2020) ELVIS-COVID-19. <https://www.ed.ac.uk/usher/elvis-covid-19/contact-us> [↑](#endnote-ref-15)
16. ClinicalTrials.gov Identifier: NCT04382131. Hypertonic saline nasal irrigation and gargling in suspected or confirmed COVID-19 (ELVIS COVID-19). <https://clinicaltrials.gov/ct2/show/NCT04382131?term=saline&cond=covid-19&draw=2&rank=6> [↑](#endnote-ref-16)
17. Kimura KS, Freeman MH, Wessinger BC et al (2020) Interim analysis of an open-label randomized controlled trial evaluating nasal irrigations in non-hospitalized patients with COVID-19. Int Forum Allergy Rhinol Sep 11 [Epub ahead of print]. <https://doi.org/10.1002/alr.22703> [↑](#endnote-ref-17)
18. ClinicalTrials.gov Identifier: NCT04347538. Impact of nasal saline irrigations on viral load in patients with COVID-19. <https://clinicaltrials.gov/ct2/show/record/NCT04347538?term=saline&cond=covid-19&draw=2&rank=1> [↑](#endnote-ref-18)
19. Delgado-Enciso I, Paz-Garcia J, Barajas-Saucedo CE et al (2020) Patient-reported health outcomes after treatment of COVID-19 with nebulized and/or intravenous neutral electrolyzed saline combined with usual medical care versus usual medical care alone: a randomized, open-label, controlled trial. Res Sq [Preprint] 10:rs.3.rs-68403. <https://doi.org/10.21203/rs.3.rs-68403/v1> [↑](#endnote-ref-19)
20. Salmon Ceron D, Bartier S et al. (2020) APHP COVID-19 research collaboration. Self-reported loss of smell without nasal obstruction to identify COVID-19. The multicenter Coranosmia cohort study. J Infect. 81(4):614-20. doi: 10.1016/j.jinf.2020.07.005 [↑](#endnote-ref-20)
21. Voshaar T. COVID-19 Therapie aus Sicht eines Aerosol-Experten. PARI.de - Artzeportal 28 Juli 2020. [https://www.pari.com/de/aerzteportal/news/covid-19-therapie-aus-sicht-eines-aerosol-experten](https://www.pari.com/de/aerzteportal/news/covid-19-therapie-aus-sicht-eines-aerosol-experten/) Accessed 10 January 2020 [↑](#endnote-ref-21)
